# Bioinspired ionic thermoreceptors with anisotropic architecture for thermotactile perception in robots

**DOI:** 10.1126/sciadv.aed5473

**Published:** 2026-05-20

**Authors:** Xuan Cai, Yilin Zeng, Pei Liu, Yifan Zhang, Linfeng Wang, Huaiyu Ke, Xue Long, Hua Jiang, Wendong Yang, Zuoxuan Gan, Shuwen Chen, Jiangjiang Duan

**Affiliations:** ^1^ Wuhan National Laboratory for Optoelectronics, School of Optical and Electronic Information, Huazhong University of Science and Technology, Wuhan 430074, China.; ^2^ Institute of Medical Equipment Science and Engineering, Huazhong University of Science and Technology, Wuhan 430074, China.

## Abstract

Emulating human skin’s ability to perceive temperature and identify material through thermotactile perception is critical for human-machine interaction, robotics operation, and prosthetic sensory feedback systems. However, conventional artificial thermal sensors are largely limited to temperature measurement and cannot replicate the thermotactile-mediated material recognition capabilities inherent in biological systems. Here, we present a bioinspired ionic thermoreceptor with anisotropic thermal response characteristics, enabling high-fidelity material recognition and accurate temperature monitoring. The device incorporates spatially specialized sensing elements that encode thermal signals into modality-specific temporal response patterns, allowing the extraction of the thermal contact coefficient for material discrimination and the absolute temperature for precise thermal monitoring. It achieves high-accuracy material recognition (98.9%) and substantial temperature resolution (0.81 millikelvins). Furthermore, robust covalent bonding between functional layers ensures mechanical durability, signal stability, and environmental resilience. This work establishes a physically grounded and energy-autonomous thermotactile sensing platform for safe, intuitive, and intelligent human-machine interaction.

## Teaser

Inspired by the human thermal system, the i-TE skin enables temperature detection and object recognition.

## INTRODUCTION

Thermotactile perception is indispensable for human-environment interaction, enabling materials recognition, hazard detection, and thermal comfort regulation (*1*, *2*). This sophisticated sensory function relies on the biological thermosensory system, in which thermoreceptors are distributed at different depths and specialized sensory pathways exhibiting distinct temporal response dynamics (*3*–*6*). Notably, rapid transient responses typically mediated by fast-conducting Aδ fibers encode heat-transfer dynamics at contact onset, whereas delayed and sustained responses primarily mediated by C fibers convey thermal sign, absolute temperature, and stimulus duration to support prolonged thermal assessment (*7*–*12*). This functional specialization allows humans to construct a comprehensive thermal perception system, supporting accurate material identification. For example, the intuitive perception that metal feels cooler than wood even when both are at identical temperatures suggests the inference of material properties through transient thermal cues.

The implementation of temporally differentiated thermal sensing strategies into artificial systems is essential for advancing thermotactile perception in next-generation prosthetics and robotics (*1*, *13*, *14*). Although artificial skins have progressed in multimodal sensing, mechanical compliance, and bioinspired signal transduction (*15*–*17*), most existing thermal sensing technologies, including ionic conductive skins (*18*–*21*), multimodal sensors (*22*–*25*), thermistor-based skins (*26*–*29*), and biomimetic thermal sensing systems (*30*, *31*), primarily focus on high-sensitivity absolute temperature detection. Consequently, the dynamic temporal response that is essential for thermotactile-mediated material recognition remains largely overlooked. Alternative material identification strategies based on optical, triboelectric, or acoustic mechanisms often introduce additional system complexity, require external power, or suffer from environmental susceptibility (*25*, *32*–*34*). Realizing an artificial thermotactile system that integrates transient thermal dynamics, environmental robustness, and energy autonomy remains profoundly challenging (*18*, *35*).

Ionic thermoelectric (i-TE) technology offers a promising route toward sensitive and self-powered thermal perception. Unlike conventional Seebeck-based electronic thermoelectric devices based on temperature gradient–inducing electron or hole diffusion (*1*, *36*), i-TE devices operate through the thermogalvanic effect, where temperature differences modulate the electrochemical potentials of reversible redox couples to generate electrical signals (*37*–*39*). The thermogalvanic effect is intrinsically reversible, providing a physical basis for bidirectional thermal-electrical transduction at soft interfaces. Specifically, under an externally applied electrical bias, the same redox processes can induce localized heating or cooling (*40*, *41*). Owing to the large entropy change associated with redox reactions, i-TE systems exhibit temperature coefficients on the order of millivolts per kelvin, substantially exceeding those of traditional thermoelectric materials (*39*, *42*), enabling high sensitivity to subtle thermotactile interactions. Moreover, their intrinsic softness and mechanical compliance allow intimate, conformal integration with complex surfaces and wearable platforms.

Here, we present a bioinspired i-TE skin with an integrated architecture designed to implement temporally differentiated thermal sensing through anisotropic thermal response pathways. By spatially configuring sensing elements with distinct thermal diffusion characteristics, the system enables rapid detection of transient heat flux at contact onset alongside stable temperature readout during sustained interaction. This architecture enables high-fidelity material recognition through transient thermal signatures while simultaneously supporting accurate temperature monitoring within a single soft platform. We demonstrate a material recognition accuracy of 98.9%, successfully distinguishing objects with nearly identical appearances and temperatures, together with real-time thermal hazard detection. Furthermore, robust covalent bonding at the hydrogel-elastomer interfaces ensures excellent mechanical resilience and environmental stability, maintaining reliable operation under repeated deformation and diverse conditions. This work establishes an integrated platform for advanced thermotactile perception in next-generation prosthetics, intelligent robotics, and human-machine interactive systems.

## RESULTS

### Bioinspired design and thermal response characteristics of the i-TE skin

**Fig. 1.**
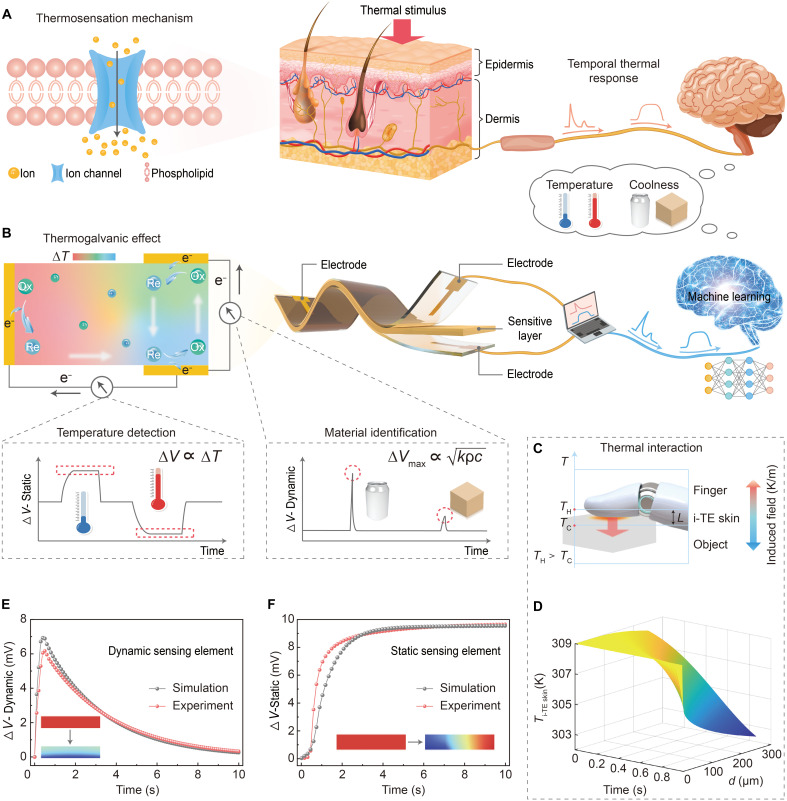
Thermotactile perception of the i-TE skin for material identification and temperature detection. (**A**) Schematic illustration of the human skin thermal perception system. (**B**) Bioinspired design concept of the i-TE skin featuring spatially separated dynamic and static sensing elements. (**C**) Thermal interaction between the i-TE skin and contacted objects. $T_{H}$ represents the temperature of the i-TE skin near the fingertip boundary, and $T_{C}$ represents the temperature at the contact interface. (**D**) Simulated temperature field of the i-TE skin in contact with an iron cube based on the Fourier heat equation. (**E**) Transient voltage response of the dynamic sensing element during contact with a steel cube, reflecting rapid heat flux–driven signaling. (**F**) Sustained voltage response of the static sensing element, encoding steady-state temperature. The insets show the corresponding heat transfer models.

The i-TE skin draws inspiration from the biological thermal perception system, where thermal information is processed through specialized sensory pathways exhibiting distinct temporal response characteristics (Fig. 1A). In biological thermosensation, rapid transient responses encode the rate of temperature change at stimulus onset, whereas sustained activity represents the steady-state temperature level (*6*–*8*). These temporally differentiated signals are transmitted to the central nervous system, enabling the perception of temperature and material properties.

Emulating these system-level principles, the i-TE skin implements anisotropic thermal response characteristics by integrating two spatially distinct sensing pathways with different thermal diffusion timescales (note S1 and fig. S1). The dynamic and static sensing elements transduce thermal stimuli into electrical signals with different temporal profiles (Fig. 1B). As a result, the device extracts two physical descriptors relevant to thermotactile interaction: the rate of heat transfer during initial contact and the equilibrium temperature during sustained interaction. Thermal-to-electrical transduction is achieved via the thermogalvanic effect in a hydrogel electrolyte containing reversible Fe^2+^/Fe^3+^ redox couples. When a temperature gradient is established across the device, the reversible redox reaction Fe^3+^ + e^−^ ⇌ Fe^2+^ proceeds asymmetrically (fig. S2), generating an open-circuit voltage increment ∆V=α×∆T, where α denotes the temperature coefficient (fig. S3) (*43*, *44*).

During contact with an object, heat transport within the i-TE skin follows spatiotemporal diffusion dynamics (Fig. 1, C and D). To analyze this process, a thermal model based on the Fourier heat equation was developed (note S2 and fig. S4). The temporal response of each sensing element is primarily determined by the Fourier number, $\mathrm{Fo}=\frac{\alpha t}{L^{2}}$ (*45*, *46*), where α is the thermal diffusivity, t is the time, and L represents the characteristic thickness. The vertically arranged dynamic sensing element, characterized by a short thermal diffusion length and a large Fourier number (*Fo* > 1.0), enables rapid heat penetration and captures rapid heat flux at contact onset, generating sharp electrical transients. The magnitude and temporal evolution of these signals are determined by the thermal contact coefficient of the contacted material, $e=\sqrt{k\rho c}$, where k, ρ, and c represent the thermal conductivity, density, and specific heat, respectively. This parameter determines the interfacial heat-extraction rate and forms the physical basis for material discrimination on the basis of transient thermal signatures (Fig. 1E). In contrast, the horizontally oriented static sensing element features a substantially longer thermal pathway (*L* > 50 mm, *Fo* < 0.05), which confines thermal perturbations near the contact interface. In this regime, the output voltage is proportional to the temperature difference between the contacted object and the reference end of the i-TE skin. Because the reference end of the i-TE skin is maintained at a nearly constant temperature, this layer provides a stable readout of absolute temperature under a quasi-equilibrium state during prolonged stimulation (Fig. 1F). The close agreement between experimental measurements and numerical simulations confirms the anisotropic thermal dynamics of the i-TE skin. It establishes the physical basis of the proposed temporal sensing mechanism.

### Fabrication and interfacial engineering of the i-TE skin

Weak interfacial adhesion between compliant polymer-based sensing layers and traditional metal electrodes has been reported to cause mechanical vulnerability and signal instability, particularly in hydrogel-based or soft ionic sensing systems (*47*, *48*). This instability originates from weak adhesion and mechanical mismatch at heterogeneous interfaces. Conventional electronic skins typically adopt stacked architectures in which a soft sensing layer is physically sandwiched between electrodes, resulting in poor mechanical integrity and susceptibility to fatigue (*49*). Recent studies indicate that covalent interfacial engineering provides more reliable routes toward mechanically robust soft devices (*50*–*52*).

**Fig. 2.**
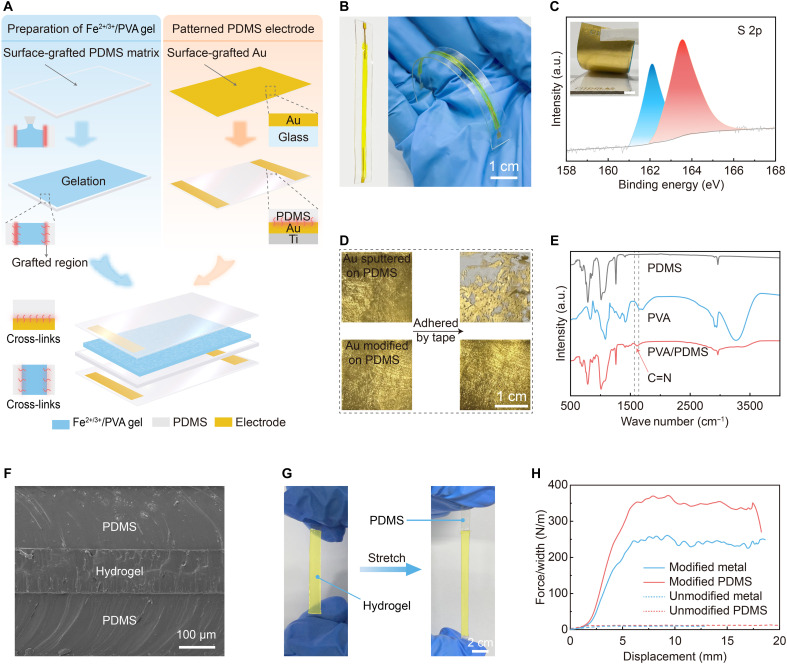
Preparation and structural characterization of the i-TE skin. (**A**) Schematic illustration of the seamlessly integrated structure of the i-TE skin. (**B**) Photograph of the i-TE skin. (**C**) S 2p x-ray photoelectron spectroscopy spectra confirming successful grafting of MPTMS on the Au surface. The inset shows the complete transfer of the MPTMS-treated Au film onto the PDMS layer. Scale bar, 5 mm. a.u., arbitrary units. (**D**) Tape-peel test comparing the adhesion strength of two types of electrodes. (**E**) Fourier transform infrared spectroscopy characterization of PVA, PDMS, and the PVA-PDMS pair. (**F**) Scanning electron microscopy image of the cross-sectional view of the i-TE skin. There is no air gap between the electrode and the Fe^2+/3+^/PVA hydrogel, which improves the stability of the wearable i-TE device. (**G**) The cross-linked PDMS-PVA gel pair maintains interfacial adhesion under mechanical deformation. (**H**) Peeling force-displacement curves of hydrogel interfaces on PDMS elastomers and metal electrodes, comparing surfaces with and without chemical modification.

To address these limitations, the i-TE skin adopts a modular integration strategy (Fig. 2A), in which the hydrogel is seamlessly encapsulated within flexible polydimethylsiloxane (PDMS) layers (Fig. 2B). This configuration is realized through a multistep interfacial engineering process that establishes strong covalent bonding across hydrogel, electrode, and elastomer interfaces. Au electrodes, deposited with a thin Ti adhesion layer, were chosen for their excellent conductivity and chemical stability under redox conditions. To further improve interfacial adhesion, the electrode surface was functionalized with a self-assembled monolayer of (3-mercaptopropyl)trimethoxysilane (MPTMS), introducing Au─S bonds, as confirmed by S 2p x-ray photoelectron spectroscopy spectra (Fig. 2C). This chemical anchoring ensures stable attachment to PDMS (fig. S5), maintaining electrode integrity and conductivity even under mechanical strain or damage (Fig. 2D and fig. S6). Meanwhile, the polyvinyl alcohol (PVA) hydrogel was selected for its tunable gelation and favorable mechanical properties. Its Young’s modulus closely matches that of PDMS and patterned electrodes, minimizing interfacial strain and mechanical mismatch (fig. S7). Given that PDMS and PVA lack a natural cross-linking ability, additional chemical modification was required (fig. S8). Specifically, the PDMS sidewalls and electrode surfaces were first plasma treated to generate reactive ─OH groups, followed by 3-aminopropyltriethoxysilane (APTES) modification and glutaraldehyde (GA) treatment. This process introduced covalent Schiff base linkages between APTES and GA, while aldehyde groups of GA reacted with hydroxyl groups of PVA (Fig. 2E), creating robust covalent coupling across all interfaces. After in situ gelation of the Fe^2+/3+^/PVA hydrogel, it was encapsulated between patterned PDMS electrode layers to form a cross-linked architecture (Fig. 2F). The Fe^2+^/Fe^3+^ redox couple remains chemically stable in acidic environments and is fully compatible with the PVA matrix (*44*, *53*), ensuring long-term electrochemical stability and mechanical integrity (table S2).

This comprehensive interfacial engineering strategy endows the i-TE skin with exceptional structural integrity and mechanical robustness. Under substantial tensile strain, the hydrogel remains firmly bonded to the PDMS substrate without observable slippage (Fig. 2G). Peel tests conducted quantitatively verified the robust adhesion achieved, yielding toughness values of 246 J m^−2^ for the metal-hydrogel interface and 366 J m^−2^ for the PDMS-hydrogel interface. Conversely, control samples relying solely on physical adhesion exhibited negligible debonding resistance and failed prematurely (Fig. 2H). These results directly demonstrate the critical role of covalent interfacial bonding in ensuring mechanical robustness and maintaining stable signal acquisition, particularly under dynamic mechanical conditions.

### Characterizations of the i-TE skin

**Fig. 3.**
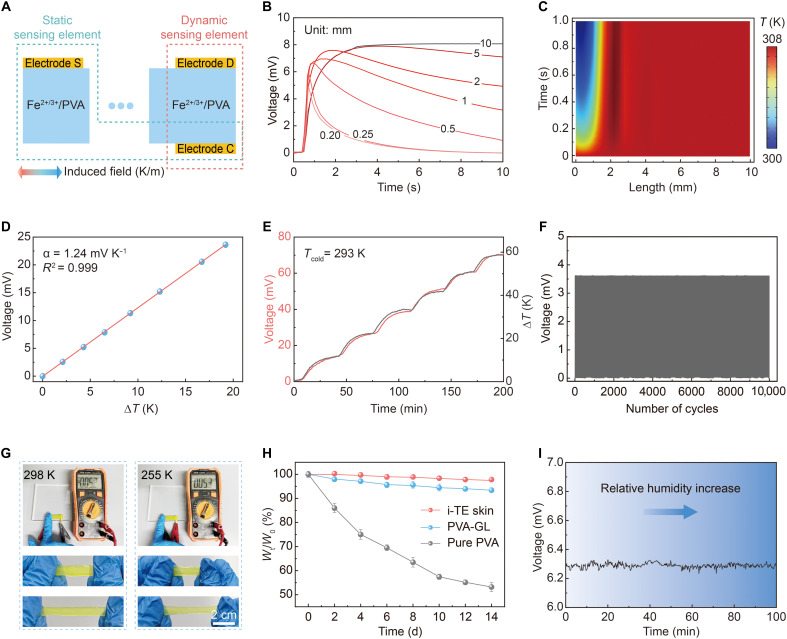
Thermoelectric performance and stability of the i-TE skin. (**A**) Schematic illustration of the temporal thermal response of the i-TE skin during thermal interaction. (**B**) Voltage-time curves of devices with different thicknesses in contact with a steel cube. (**C**) Temporal evolution of the temperature across the i-TE skin during thermal interaction with a steel cube. (**D**) Temperature coefficient of the i-TE skin under different temperature gradients. (**E**) Voltage-time evolution curve during heating processes. The temperature range is 60 K, with the cold end at 293 K. (**F**) Response of the sensor during cyclic contact with glass at room temperature. (**G**) Antifreeze performance of the hydrogel electrolyte. (**H**) Normalized weight change of the i-TE skin over 2 weeks in air (data are the means ± SD, *n* = 3). d, days. (**I**) Output voltage of the i-TE skin under different relative humidity conditions. The humidity range is from 40 to 80%.

The temporal thermotactile response of the i-TE skin is governed by thickness-controlled thermal diffusion. As illustrated in Fig. 3A, the propagation of thermal disturbances occurs more rapidly in the dynamic sensing element compared to the static sensing element. Along the vertical dynamic pathway, heat flux decays rapidly, enabling a transient response of interfacial heat transfer during initial contact. In contrast, the lateral diffusion along the horizontal static pathway occurs more slowly, controlling sustained temperature readout. The stable thermal conductivity (κ) of the device facilitates efficient establishment of temperature gradients required for thermotactile sensing (note S3 and fig. S9). While shorter conduction paths accelerate the response, excessively thin designs compromise signal stability and mechanical robustness (Fig. 3B). Simulations indicate that thermal perturbations at the contact interface diffuse across the sensing layer in ~0.4 s, corresponding to a diffusion length below 0.5 mm (Fig. 3C). To balance response speed, signal fidelity, and mechanical durability, the i-TE skin thickness was optimized to 250 μm. Under typical contact durations (<0.5 s), this geometry yields a Fourier number *Fo* ≈ 0.8, placing the dynamic sensing element within a transient heat flux–dominated regime. This configuration enables efficient capture of material-dependent thermal transients while maintaining stable voltage output and robust mechanical performance.

The i-TE skin demonstrates excellent thermoelectric performance enabled by the Fe^2+^/Fe^3+^ redox couple. As evaluated with a dedicated setup (fig. S10), the open-circuit voltage scales linearly with temperature gradient, exhibiting a temperature coefficient of 1.24 mV K^−1^ with *R*^2^ = 0.999 (Fig. 3D). The response closely matches that of a commercial thermocouple, exhibiting 99.9% linearity across the tested range (fig. S11). The i-TE skin operates stably over a 60 K temperature range (Fig. 3E), resolves temperature variations as small as 0.01 K (fig. S12), and achieves a noise-limited resolution of 0.81 mK. Its strong resistance to motion-induced noise during human movement (fig. S13) enables reliable thermal signal acquisition for artificial tactile sensing.

Beyond intrinsic sensing performance, the i-TE skin exhibits substantial durability and environmental resilience. It maintains stable voltage output over 10,000 contact-separation cycles (Fig. 3F) and withstands 10,000 stretching-release cycles at 30% strain without degradation (fig. S14). The output voltage is unaffected by applied pressure (fig. S15), confirming the mechanical robustness required for robotic and prosthetic applications. Considering that ion transport within the hydrogel relies on an aqueous medium, antifreeze and dehydration resistance were further engineered. Glycerol (GL) was introduced to depress the freezing point, ensuring that the hydrogel maintains its flexibility and ionic conductivity under subzero conditions (Fig. 3G). In addition, full encapsulation and the intrinsic dehydration resistance of the hydrogel limited water loss by just 3% after 15 days in ambient air (Fig. 3H), while voltage output remained stable across varying humidity levels (Fig. 3I). Accelerated aging tests at 60°C and 40% relative humidity for 72 hours showed no measurable mass loss or sensitivity degradation (fig. S16), supporting reliable long-term operation in wearable and robotic applications.

### Thermotactile perception performances of the i-TE skin

To evaluate the thermotactile-mediated materials recognition, nine common materials with distinct thermal properties (table S1) were systematically tested under controlled ambient conditions (298 K). The temperature of the i-TE skin was set at 309 K, closely approximating that of the human body temperature before contact. Foam insulation and a sealed enclosure were used to minimize heat convective exchanges (fig. S17). Under these conditions, radiative and convective heat losses (~10^2^ W/m^2^) are orders of magnitude smaller than the initial conductive flux across the contact interface (~10^4^ to 10^5^ W/m^2^) and thus do not influence the transient response used for analysis. The temperature evolution derived from the i-TE skin closely matched that obtained using commercial thermocouples, with only minor deviations attributable to interfacial thermal resistance and temporal response delays (fig. S18). This agreement confirms the reliability of the i-TE skin in thermal sensing as a basis for material identification on the basis of transient thermal responses.

**Fig. 4.**
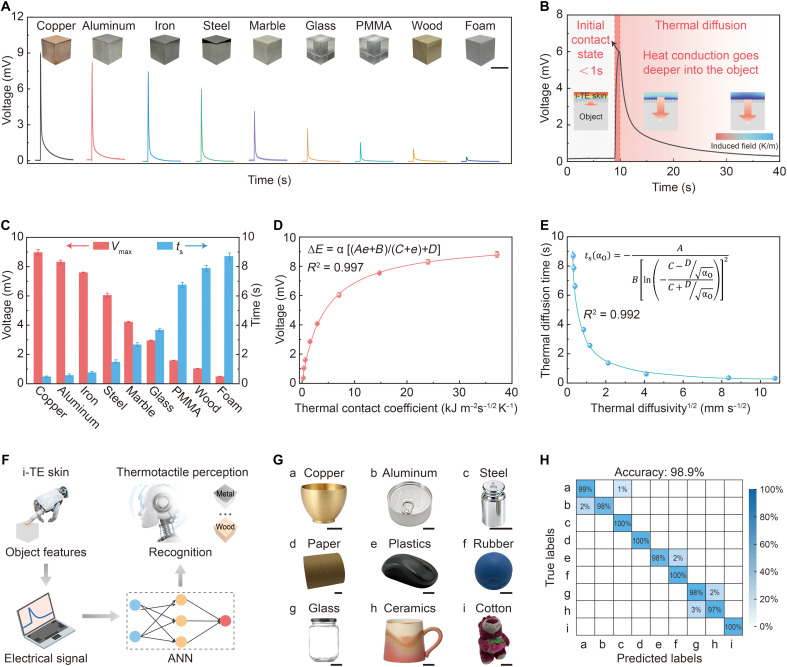
Evaluation of the thermotactile perception performance of the i-TE skin. (**A**) Typical voltage-time responses of the i-TE skin to different materials (means ± SD, *n* = 5; scale bar, 50 mm). PMMA, polymethyl methacrylate. (**B**) Representative voltage response from a dynamic sensing element during contact with a steel object, highlighting the transient material-dependent response. The inset schematically illustrates the heat-transfer dynamics. (**C**) Characteristic parameters extracted from the transient response curves. (**D**) Correlation between the peak voltage and the thermal contact coefficient of each material (means ± SD, *n* = 5). (**E**) Relationship between thermal diffusivity and thermal diffusion time (means ± SD, *n* = 5). (**F**) Schematic illustration of the machine learning–assisted thermotactile material recognition workflow, including transient signal acquisition, feature extraction, dataset construction, ANN training, and classification output. (**G**) Nine commercially available objects used in the robotic thermotactile recognition experiments. The photograph was captured under laboratory conditions. Scale bar, 25 mm. (**H**) Confusion matrix of the nine tested objects, showing a recognition accuracy of 98.9%.

As shown in Fig. 4A, the voltage-time curves exhibited distinct, material-specific transient signatures. These signatures originate from a dynamic thermoelectrochemical response during the initial contact phase. Upon contact, rapid interfacial heat exchange induces a sharp voltage spike, followed by a decay governed by heat diffusion into the contacted material (Fig. 4B). Materials with higher thermal contact coefficients, such as copper and aluminum, induce more intense and faster transients than materials with lower thermal contact coefficients, such as wood and plastic (fig. S19). The resulting dynamic voltage signals exhibited a strong linear correlation with temperature evolution (*R*^2^ > 0.99; fig. S20) and remained distinguishable under varying contact pressures (fig. S21). In addition, the transient responses were independent of contact frequency (fig. S22), suggesting that shorter contact durations can accelerate the identification process without sacrificing accuracy. Reliable material discrimination was achieved with contact durations as short as 0.4 s (fig. S23). Unlike conventional heat-flux sensors designed for quantitative measurement of steady-state heat flux under controlled conditions, the i-TE skin is specifically engineered for thermotactile interaction sensing, where transient thermal exchange at contact onset serves as the primary information carrier (table S3). To characterize these dynamics, key temporal features were extracted from the transient responses (Fig. 4C), including peak amplitude (*V*_max_), thermal diffusion timescale (*t*_s_), and signal slope (detailed in note S2). These features encode rich, material-specific thermal information. As shown in Fig. 4D, the peak voltage exhibited an excellent correlation with the material’s thermal contact coefficient (*R*^2^ = 0.997). Similarly, a strong inverse correlation was found between material thermal diffusivity and diffusion time (*R*^2^ = 0.992; Fig. 4E). These results validate that the transient thermotactile responses of the i-TE skin effectively reflect intrinsic material thermal properties through their temporal characteristics.

The i-TE skin demonstrated material identification based solely on thermal cues. To enable high-fidelity recognition, machine learning was incorporated into the thermotactile sensing framework (Fig. 4F). Nine distinct objects used in the robotic grasping experiments were equilibrated at room temperature before testing (Fig. 4G). The i-TE skin was brought into contact with each object for 100 repeated trials, during which transient temperature evolution was recorded to capture interfacial heat-exchange dynamics. A robotic arm was programmed to grasp the nine objects sequentially, yielding a total of 900 valid thermotactile datasets. Each dataset corresponded to a single contact event and consisted of transient thermal features extracted from the dynamic sensing element. These datasets were used exclusively for offline supervised learning, in which an artificial neural network (ANN) was trained using an 8:1:1 split for training, validation, and testing to avoid overfitting (fig. S24). The trained model achieved a material recognition accuracy of 98.9% (Fig. 4H), demonstrating robust material classification based on thermotactile signatures.

### Application of thermotactile systems in real-world scenarios

**Fig. 5.**
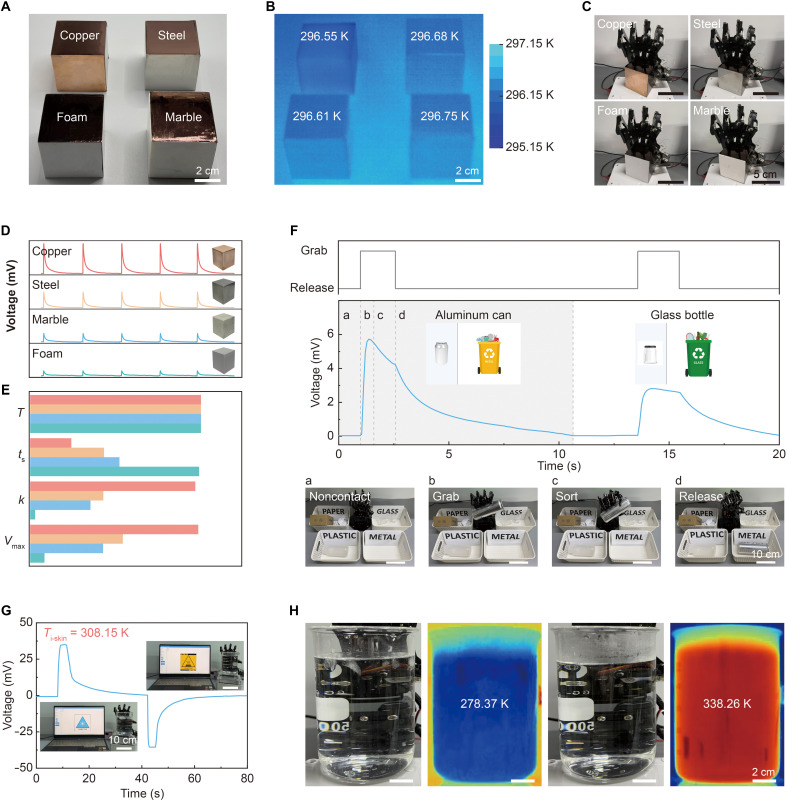
Practical application of the intelligent robot system with the i-TE skin. (**A**) Physical images of four representative objects. (**B**) Corresponding infrared thermograms of objects. (**C**) Real-time recognition of copper, stainless steel, marble, and foam. (**D**) Voltage signal responses to different objects. (**E**) Key parameters extracted from the temporal voltage signals. (**F**) Dynamic voltage signals during two consecutive waste-sorting processes for recognizing an aluminum can and a glass bottle. The inset highlights the feedback signal during grasping. The lower panel shows the classification process for the aluminum can. The sequential stages are labeled as a to d, corresponding to noncontact (a), grasping (b), classification (c), and release (d). (**G**) Voltage responses of the i-TE skin upon contact with hot and ice water. The inset shows the feedback effect of a thermal injury stimulus. (**H**) Experimental scenarios of the i-TE skin contacting hot and ice water, along with corresponding infrared thermographic images.

The i-TE skin can infer the intrinsic thermal properties of the objects on the basis of transient responses via the thermogalvanic effect, providing a robust and scalable platform for material recognition. Four visually similar materials, including copper, stainless steel, marble, and foam, were selected for validation (Fig. 5A) and uniformly coated to eliminate optical cues (fig. S25). Unlike vision-based methods that fail to differentiate objects with similar appearances or infrared thermography that showed negligible temperature differences among the samples (<0.2 K; Fig. 5B), the i-TE skin generated distinct thermoelectrical responses upon contact (Fig. 5C). This allowed for the extraction of material-specific thermal fingerprints (Fig. 5D and movie S1). When integrated into a robotic fingertip, the i-TE skin continuously converts contact-induced thermal signals into electrical signals (fig. S26). The signals are first filtered and denoised, then processed by a microcontroller unit, and passed to a pretrained cascade classifier for real-time material recognition (fig. S27). The system analyzed three key parameters, including voltage peak, rate of change, and thermal diffusion time, to construct a material thermal response feature vector for each contact event, enabling accurate discrimination of materials with similar appearance and temperature (Fig. 5E). The i-TE skin enables the concurrent extraction of transient material-specific signatures and absolute temperature information within a single brief contact, ensuring robust material recognition under varying object temperatures (fig. S28).

To demonstrate its practical utility, we developed an intelligent robotic waste-sorting system equipped with the i-TE skin. This system successfully identified common recyclable items, such as an aluminum can and a glass bottle, on the basis of their unique thermal responses. The aluminum can, with a high thermal contact coefficient, exhibits rapid heat transfer, reaching a peak voltage of 5.89 mV within 0.5 s. In contrast, a glass bottle, with a lower thermal contact coefficient, produced a peak voltage of only 2.93 mV with a response delay exceeding 1.0 s (Fig. 5F). Including communication latency, the entire sorting process was completed within 5.0 s.

Beyond material recognition, the i-TE skin also provides real-time safety monitoring for human-machine interactions. On the basis of human thermal pain thresholds [318 K for an overly hot state (*54*) and 278 K for a supercooled state (*55*)], we implemented a dual-threshold warning mechanism. If an object’s temperature exceeds these safety limits, the system immediately triggers an alert and halts robot operation (Fig. 5, G and H). This closed-loop feedback mechanism simulates the protective reflex response of the human body. With a rapid response, high sensitivity, and stable voltage output, the i-TE skin ensures safe and reliable operation, expanding the practical application scope of thermotactile technology in robotics and intelligent systems.

## DISCUSSION

The i-TE skin provides a versatile platform for advanced thermotactile sensing that extends beyond the capabilities of conventional temperature sensors and heat-flux sensors. Unlike most electronic skins that provide only steady-state temperature readouts, this system integrates spatially specialized sensing elements with anisotropic thermal-response characteristics that exhibit modality-specific response patterns. This design enables concurrent encoding of transient heat-transfer dynamics and steady-state temperature information, thereby supporting accurate material discrimination based on thermal contact coefficients and precise temperature monitoring within a single soft device. The device further demonstrates excellent mechanical durability and environmental stability, maintaining reliable performance under repeated deformation and varied operating conditions. When integrated with a robotic hand, the i-TE skin enables real-time material identification, automated classification, and thermal hazard detection, highlighting its applicability to prosthetics, wearable devices, and intelligent robotic systems that require reliable and adaptive thermal feedback.

Beyond passive sensing, the intrinsic reversibility of the thermogalvanic effect allows the i-TE skin to operate in an active mode. Under an applied electrical bias, the device can generate localized heating or cooling, enabling controllable thermal modulation at the interface. This capability establishes a direct physical pathway toward active thermotactile interfaces. Such interfaces are gaining traction, as recent studies demonstrate that controlled heat flux can reproduce material-dependent thermal sensations in immersive virtual reality/augmented reality environments. Prospectively, integrating active thermal modulation with material-aware thermal sensing may enable closed-loop, adaptive, and context-sensitive human-machine interaction systems.

## MATERIALS AND METHODS

### Materials

All chemical reagents were sourced from Aladdin Bio-Chem Technology Co., Ltd. (China), and used without further purification. Water used in all experiments was ultrapure, obtained through a Milli-Q system (Simplicity, Millipore, France).

### Methods

#### Preparation of hydrogels

First, 1.2 g of PVA, 0.099 g of FeCl_2_·4H_2_O, 0.135 g of FeCl_3_·6H_2_O, and 0.12 g of LiCl in an 8:2 mixture of 8.8 ml of water and glycerin were stirred magnetically at 90°C for 6 hours to create a homogeneous solution. After cooling the solution to 50°C, 0.8 ml of H_3_PO_4_ was added and continued stirring for 1 hour. A homogeneous solution of Fe^2+/3+^/PVA/H_3_PO_4_ precursor was formed for subsequent experiments.

#### Fabrication of stretchable electrodes and sensors

*Preparation of the PDMS-based flexible electrode.* A customized shadow mask was securely fixed onto a glass substrate, and a 150-nm patterned Au film was deposited via magnetron sputtering (JCP-600M4). To modify the Au film, a treatment solution of (3-mercaptopropyl)trimethoxysilane (95% purity), ethanol, and deionized water in a 1:4.5:4.5 volume ratio was prepared. The pH of the solution was adjusted using glacial acetic acid (99.5% purity) at a 1:20 volume ratio of acetic acid to (3-mercaptopropyl)trimethoxysilane. The glass slide with the patterned Au film was immersed in this solution for 10 min. PDMS (Sylgard-184, Dow Corning) was mixed at a base–to–cross-linker weight ratio of 10:1 and degassed in a vacuum oven. The PDMS precursor was spin coated onto the treated Au film and cured at 60°C for 6 hours. The resulting Au/PDMS flexible electrode was then carefully detached from the glass substrate. Then, the electrode was sputtered with Ti.

##### Assembly of the sensor structure

The A and B components of PDMS were mixed in a 10:1 ratio and cured at 60°C overnight to form a 200-μm intermediate PDMS layer. A rectangular groove (150 mm in length and 1.0-mm hole spacing) was carved into this PDMS layer using a scalpel, with small holes reserved for injecting hydrogel precursor solutions. The sidewalls of the grooves and the Au/PDMS electrode were treated with oxygen plasma (PC5-Plus) at 40 W for 60 s. After plasma treatment, these layers were gently stacked layer by layer, forming a sensor container that housed Fe^2+/3+^/PVA sensing elements.

*Chemical modification of PDMS and electrodes.* To achieve robust integration, the Au/PDMS-PDMS-Au/PDMS container was immersed in a solution containing 10 vol % APTES (99% purity), 45 vol % ethanol, and 45 vol % deionized water at 60°C for 10 min. The PDMS layers were subsequently rinsed with ethanol and deionized water. The aminated container was further treated in a 5.0 vol % aqueous solution of GA (50 vol % in water) at 60°C for another 10 min, followed by additional rinsing with ethanol and deionized water.

*Gelation and sealing.* The Fe^2+/3+^/PVA solution was injected into the grooves using a syringe. This facilitated covalent cross-linking between the PDMS chains and PVA chains. After the hydrogel precursor was initially cured for 10 min, the injection port was sealed to complete the preparation of the overall structure.

#### Morphology and composition characterization

The sensor underwent freeze-drying followed by snap-fracturing using liquid nitrogen to preserve its internal microstructure. The fractured samples were characterized using a field emission scanning electron microscope (Sirion 200, FEI) to examine the morphology at a high resolution. To assess the chemical interactions and confirm covalent bonding between PDMS and the Fe^2+/3+^/PVA hydrogel, Fourier transform infrared spectroscopy (Nicolet iS50R, Thermo Fisher Scientific) was performed. All optical photographs were captured by the authors using an iPhone.

#### Electrical performance characterization

The thermogalvanic effect of Fe^2+/3+^ redox species was evaluated using a planar device configuration. The open-circuit voltage (*V*_oc_) was measured with a Keithley 2400 multimeter, and the temperature gradient across the device was monitored using a thermocouple data logger (USB-TC-08, Pico Technology, St. Neots, UK). Data points were collected at a voltage scanning rate of 0.1 s per point.

The resistance of the Au/PDMS electrodes was tested under bending and stretching conditions. Measurements were conducted using a Keithley 2400 multimeter, allowing for the evaluation of electrode stability and performance during deformation.

#### Experimental validation and theoretical model of anisotropic heat transfer

The i-TE skin was pre-equilibrated to 36°C and then brought into contact with a steel block maintained at 25°C. Voltage outputs from the dynamic (vertical) and static (horizontal) sensing elements were recorded independently using their respective electrode pairs.

Transient heat transfer was modeled using COMSOL Multiphysics. The i-TE skin and contacted object were assigned uniform but different initial temperatures. Perfect thermal contact was assumed at the interface to isolate intrinsic heat-transfer dynamics, while all other boundaries were treated as thermally insulated to suppress convective and radiative losses.

#### Mechanical property characterization

Samples were prepared in a dumbbell shape for tensile testing. The mechanical properties were tested using a mechanical testing machine (RWT10, REGER) at a constant strain rate of 25 mm/min. For interfacial toughness measurements, Sil-Poxy silicone adhesive was used to bond a 100-μm-thick polyethylene terephthalate film to the test surface, providing a cushion layer. The peel pairs, each with a thickness of 200 μm and plasma treated, were tested at a peel rate of 50 mm/min. The specimen width is 2.5 cm. Control samples are obtained by directly applying hydrogels to PDMS and metal surfaces.

#### Signal test of the contact process between the sensor and the object

The sensor measurement setup comprises a heating system, a single-degree-of-freedom linear actuator, and a sensor. The linear actuator and heater are powered by a direct current (dc) power source. A constant current is supplied to the heater to elevate the sensor temperature to a stable range of 36 ± 0.5°C, which is close to human skin’s temperature. The test objects are stabilized at room temperature (25°C) for 2 hours to ensure that all objects have the same temperature. The sensor makes contact with the material using a controlled target force of 3 N. Direct thermal contact between the sensor and the test object causes temperature changes within the sensor body, facilitating material identification. To mitigate heat transfer from the heater to the actuator, which could potentially affect sensor performance, a foam layer is strategically placed between them. During measurements conducted at room temperature, a constant force is applied by the linearly controlled actuator to touch and release the test object. Simultaneously, the K2400 data acquisition system captures output voltage readings. The entire test system is isolated from the surrounding environment by foam panels.

#### Measurement for object recognition

For the collection of signals related to object recognition, a sensor is attached to one end of a robotic arm. The heating module is powered by a 1.2-V dc power supply to heat it to a temperature of 35°C. In this experiment, the initial temperature of the sensor is set to 35°C (approximately human skin’s temperature), while the temperature of all objects being tested is 25°C (room temperature). The robotic arm is programmed to grasp and release the objects under test.

#### Temperature calibration of the i-TE skin

After reaching thermal equilibrium, the temperature readings from the i-TE skin and a reference thermocouple are simultaneously recorded at each temperature point. To improve measurement accuracy, each is repeated three times. The average temperature measured by the thermocouple is used as the *x*-axis value, while the corresponding average value from the i-TE sensor is assigned to the *y* axis. A scatterplot is then generated, and a calibration curve is obtained via linear regression. The resulting fitting equation is used to determine the calibration coefficient for accurate temperature mapping.

#### Machine learning for material recognition

To evaluate the material recognition capability of the i-TE skin, time-domain feature extraction was combined with ANN-based classification. Recognition experiments were performed on nine materials with distinctly different thermal properties.

#### Offline dataset construction and ANN training

For controlled material identification, the i-TE skin was brought into repeated contact with each material under identical conditions. The nine tested objects were commercially available common materials arranged for demonstration of the robotic grasping experiments. Each contact constituted one complete trial, during which the transient voltage signal was recorded. From each recorded signal, three characteristic time-domain features were extracted: (i) voltage peak, (ii) initial rising slope, and (iii) time to reach the peak. Together, these features form a compact thermal-response vector that captures both the magnitude and temporal dynamics of the heat-transfer process.

Each material was tested 100 times, yielding a total of 900 valid samples. The dataset was randomly divided into training, validation, and testing subsets with a ratio of 8:1:1 for model training, hyperparameter tuning, and performance evaluation, respectively.

An ANN was used for multiclass material classification. The network architecture consisted of an input layer with 12 neurons, followed by four hidden layers with 32, 128, 64, and 32 neurons, respectively, using rectified linear unit activation functions. A softmax-activated output layer was used for material prediction. The model was trained using the Adam optimizer (learning rate, 0.0005) with categorical cross-entropy loss, and early stopping was applied to prevent overfitting. Classification performance was evaluated using accuracy, mean squared error, and confusion matrices. All data processing and model implementation were carried out in Python.

#### Online real-time inference during operation

After offline training, the ANN parameters were fixed and deployed for real-time material recognition. During real-time operation, material identification is performed on a per-contact-event basis rather than relying on repeated trials or batch processing. When the i-TE skin contacts a material surface, a single transient voltage response is generated. From this single response, the voltage peak, rising slope, and time to peak are extracted in real time and immediately fed into the pretrained ANN, which outputs one material classification result corresponding to that contact event.

In this framework, repeated contact trials are used exclusively for offline dataset construction and model training and do not constrain real-time operation. The ANN output is event-driven, with one classification result generated per contact. This workflow enables real-time material recognition during tactile interaction while maintaining robust classification performance. The same feature extraction and real-time ANN inference pipeline was used in the waste-sorting demonstration, where each contact event directly produced a classification output for robotic sorting.

## Supplementary Materials

### The PDF file includes:

Notes S1 to S4 Figs. S1 to S28 Tables S1 to S4 Legend for movie S1 References
