# Supplementary material for: Bioinspired ionic thermoreceptors with anisotropic architecture for thermotactile perception in robots
Source: Sci Adv. 2026 May 20;12(21):eaed5473. doi: 10.1126/sciadv.aed5473 (PMC13189106; doi:10.1126/sciadv.aed5473)
Supplement: Supplementary file 1 — Notes S1 to S4 Figs. S1 to S28 Tables S1 to S4 Legend for movie S1 References [file sciadv.aed5473_sm.pdf]

Science Advances
AAAS

Supplementary Materials for

Bioinspired ionic thermoreceptors with anisotropic architecture for thermotactile perception in robots

Xuan Cai *et al.*

Corresponding author: Shuwen Chen, shuwenchen@hust.edu.cn; Jiangjiang Duan, jiangjduan@hust.edu.cn

Sci. Adv. **12**, eaed5473 (2026)
DOI: 10.1126/sciadv.aed5473

The PDF file includes:

Notes S1 to S4
Figs. S1 to S28
Tables S1 to S4
Legend for movie S1
References

Other Supplementary Material for this manuscript includes the following:

Movie S1

Note S1. Operation mechanism of the i-TE skin during thermal interaction

The i-TE skin operates as a thermogalvanic sensing system that converts localized thermal stimuli into electrical signals through temperature-dependent electrochemical potentials. As illustrated in fig. S1a, the device consists of a contact electrode (Electrode C), a proximal electrode (Electrode D), a distal electrode (Electrode S), and an intervening hydrogel electrolyte containing reversible redox couples. By deliberately configuring the spatial separation between electrodes, the system forms two functionally distinct sensing elements: a dynamic sensing element (Electrode C–D) with an ultrashort ionic pathway, and a static sensing element (Electrode C–S) connected through a substantially longer hydrogel path.

Thermogalvanic effect

The operation of the i-TE skin is governed by the thermogalvanic effect. For a redox reaction, Ox + ne^- ⇋ Red, its electrode potential (E) is obtained by the Nernst equation:

$$E = E^0 + \frac{RT}{nF} ln \frac{a_\mathrm{O}}{a_\mathrm{R}}$$

Here, E^0 represents the standard electrode potential, R is the ideal gas constant, T is the temperature at which the redox reaction occurs, n is the number of charge transfers in the redox reaction, F is the Faraday constant, and a_O and a_R are the activities of oxides and reducents, respectively.

In a typical thermogalvanic cell comprising two identical electrodes immersed in a redox electrolyte, an imposed temperature gradient induces asymmetric interfacial redox reactions at the hot and cold electrodes, resulting in a measurable potential difference. For Fe^{3+}/Fe^{2+} redox couples, at the cold electrode, oxidation of Fe^{2+} releases electrons, while reduction of Fe^{3+}occurs at the hot electrode, sustaining continuous electron flow in the external circuit.

Encoding of thermal interaction into electrical signals

Because the electrode potential is directly determined by the local interfacial temperature, the voltage measured between the contact electrode and either the proximal or distal electrode inherently encodes the thermal state of the system. When the i-TE skin comes into contact with an object, rapid interfacial heat transfer causes an abrupt temperature change at the contact electrode (fig. S1b–c).

In the dynamic sensing element (Electrode C–D), the short thermal diffusion length and small effective thermal mass enable rapid propagation of this thermal disturbance to the proximal electrode. As a result, a transient voltage signal is generated immediately upon contact (Fig. 1E). The amplitude and temporal evolution of this transient response are governed by the rate of heat transfer at the interface and therefore reflect the object's thermal contact coefficient. This transient signal captures material-specific thermal cues during the initial contact stage.

In contrast, the static sensing element (Electrode C–S) is characterized by a much longer ionic and thermal pathway. Heat transfer along this path is strongly attenuated owing to the larger effective thermal mass and lateral thermal extension of the hydrogel. Consequently, the distal electrode remains close to thermal equilibrium during short-time contact, and the resulting voltage primarily reflects the quasi-steady-state temperature difference between the contacted object and the distal electrode (Fig. 1F). This configuration enables stable and reliable measurement of absolute temperature during sustained thermal interaction.

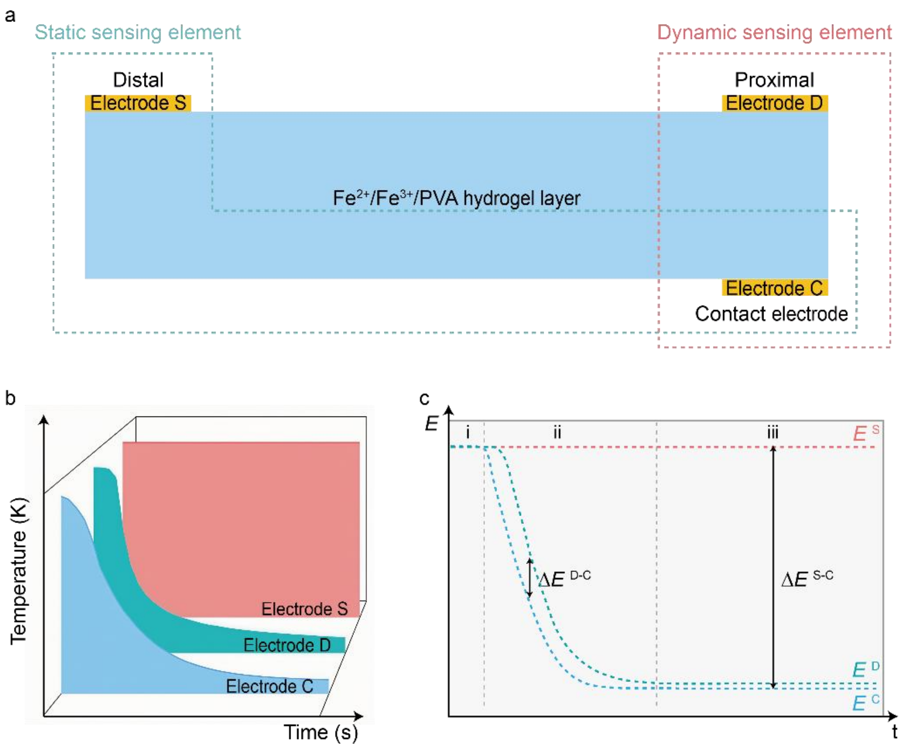

Figure S1. Schematic of the operation mechanism of the i-TE skin. a) Structural diagram of the i-TE skin. b) Temperature evolution of the electrodes during thermal interaction. c) Corresponding thermopotential response of the electrodes during thermal interaction.

Note S2. A simplified physics-informed model of sensor–object thermal interaction

Thermal modeling of sensor–object contact provides an essential physical framework for interpreting transient temperature responses during thermo-tactile interactions *(56,57)*. In this work, we employ a simplified, physics-informed analytical model to elucidate the dominant heat-transfer mechanisms governing the dynamic response of the i-TE skin. Importantly, the purpose of this model is not to deliver a fully predictive thermophysical description or to extract absolute material parameters. Rather, it is designed to capture the key trends of short-time transient heat transfer under practical tactile conditions and to guide feature extraction from experimentally measured signals.

Model description and physical assumptions

The i-TE skin adopts a sandwich architecture consisting of two electrodes separated by an ionic electrolyte layer containing reversible redox couples. During thermal contact, the outer electrode serves as the sensor–object interface, while the inner electrode is thermally anchored to a reference heat source. The electrolyte layer, with a thickness of approximately 0.2 mm, mediates both heat conduction and thermogalvanic signal transduction.

To render the problem analytically tractable while retaining physical relevance, the sensor is modeled as a single finite-thickness layer with uniform thermophysical properties, such as thermal conductivity, density, and specific heat (k_{s}, ρ_{s}, c_{s}). The external object is treated as a semi-infinite solid characterized by constant properties (k_{o}, ρ_{o}, c_{o}), and an initial uniform temperature T_{o0} is the initial temperature.

Prior to contact, the temperature distribution within the electrolyte layer is assumed to vary linearly with position along the spatial coordinate x, as illustrated in fig. S4. The temperature profile can be expressed as:

$$T_{\mathrm{s}}^{(\mathrm{i})} = T_{\mathrm{s}}^{(\mathrm{i})}(x) = T_{\mathrm{H}} + (T_{\mathrm{s0}} - T_{\mathrm{H}})\frac{x}{L} \quad (1)$$

where T_{H} is the constant temperature, T_{s0} is the initial temperature at the contact end of the sensor (at $x = L$), with $T_{\mathrm{H}} > T_{\mathrm{s0}}$, and L is the thickness of the electrolyte layer.

Governing equations and boundary conditions

Upon contact, the heat-transfer process is described as a one-dimensional transient conduction problem involving a finite-thickness sensor layer coupled to a semi-infinite object.

The mathematical problem is now well-posed: the Fourier heat equation can be written for the two temperature fields associated with the sensor $T_{\mathrm{s}}(x, t)$.

A schematic diagram of the model is presented in fig. S4. Specifically, the governing equations are:

$$\frac{\partial^2 T_{\mathrm{s}}}{\partial x^2} = \frac{1}{\alpha_{\mathrm{s}}}\frac{\partial T_{\mathrm{s}}}{\partial t}, \begin{cases} T_{\mathrm{s}}(x = 0, t) = T_{\mathrm{H}}, t \geq 0 \\ T_{\mathrm{s}}(x, t = 0) = T_{\mathrm{s}}^{(\mathrm{i})}(x), 0 \leq x \leq L \end{cases} \quad (2)$$

$$\frac{\partial^2 T_{\mathrm{o}}}{\partial x^2} = \frac{1}{\alpha_{\mathrm{o}}}\frac{\partial T_{\mathrm{o}}}{\partial t}, \begin{cases} T_{\mathrm{o}}(x \to \infty, t) = T_{\mathrm{o0}}, t \geq 0 \\ T_{\mathrm{o}}(x, t = 0) = T_{\mathrm{o0}}, x \geq L \end{cases} \quad (3)$$

with interfacial continuity conditions

$$\begin{cases} T_s(x=L,t) = T_o(x=L,t), t \geq 0 \\ k_s\left(\frac{\partial T_s}{\partial x}\right)_{x=L} = k_o\left(\frac{\partial T_o}{\partial x}\right)_{x=L}, t \geq 0 \end{cases} \tag{4}$$

$$\alpha = \frac{k}{\rho c} \tag{5}$$

where T represents temperature, α represents thermal diffusivity, and t represents time. The subscripts s and o denote the thermoelectric hydrogel sensor and the object, respectively. The superscript i indicates initial conditions.

Analytical solution and interpretation

We report in the following the exact solutions of the Partial Differential Equation for the temperature fields obtained according to the Laplace transform approach. The temperature fields of the sensor can be expressed as:

$$T_s(x,t) = \sum_{n=1}^{\infty} \frac{\left[-k_s(T_{s0}-T_H)-k_o\sqrt{\frac{p_n}{\alpha_o}}(T_{s0}-T_{o0})L\right]\sinh\left(\sqrt{\frac{p_n}{\alpha_s}}x\right)}{2L\cdot p_n\cdot D(p_n)} e^{p_n t} + T_s^{(i)} \tag{6}$$

$$k_s\sqrt{\frac{p_n}{\alpha_s}}\cosh\left(\sqrt{\frac{p_n}{\alpha_s}}L\right) + k_o\sqrt{\frac{p_n}{\alpha_o}}\sinh\left(\sqrt{\frac{p_n}{\alpha_s}}L\right) = 0 \tag{7}$$

where p_n is the root of the characteristic equation (7), and $D(p_n)$ is the derivative of the denominator at p_n.

This solution consists of a steady-state linear distribution and a transient attenuation series, and depicts the change process of the sensor over time and space.

The thermal contact coefficient (e) is defined as the geometric mean of thermal conductivity and volumetric heat capacity. In formula:

$$e = \frac{k}{\sqrt{\alpha}} \tag{8}$$

Combining the above formulas (6) and (8), the sensor temperature response can be expressed as a rational function of the thermal contact coefficient of the object:

$$T_s(e_o) = \frac{(\mathrm{A}\cdot e_o + \mathrm{B})}{(e_o + \mathrm{C})} + \mathrm{D} \tag{9}$$

Where,

$$\begin{cases} \mathrm{A} = T_{s0} - T_{o0} \\ \mathrm{B} = e_s(T_{s0} - T_H)\frac{L-x}{L} \\ \mathrm{C} = e_s \\ \mathrm{D} = T_s^{(i)} \end{cases} \tag{10}$$

This is a rational function that depends on e_o. In this case, we will use a nonlinear fitting algorithm to estimate the values of the parameters A, B, C, and D.

It can be seen from formula (6) that as time t increases, $T_s(x,t)$ will gradually tend to be quasi-stable. Assume that when $t = t_s$, t_s is denoted as the thermal diffusion time, and the thermal process enters the quasi-steady state.The formula (6) will be fitted as follows:

$$t_{\mathrm{s}}(\alpha_{\mathrm{o}}) = -\frac{\mathrm{A}}{\mathrm{B}\left[\ln\left(-\frac{\mathrm{C}-\mathrm{D}/\sqrt{\alpha_{\mathrm{o}}}}{\mathrm{C}+\mathrm{D}/\sqrt{\alpha_{\mathrm{o}}}}\right)\right]^2} \tag{11}$$

Where,

$$\begin{cases} \mathrm{A} = 4L\ln 0.05 \\ \mathrm{B} = \alpha_{\mathrm{s}} \\ \mathrm{C} = k_{\mathrm{s}} \\ \mathrm{D} = k_{\mathrm{o}}\sqrt{\alpha_{\mathrm{s}}} \end{cases} \tag{12}$$

This model only considers the most fundamental factors in the heat transfer process, namely the initial temperatures and thermal properties of the sensor and the material. Despite its limitations in certain aspects, the existing model still provides a preliminary approximation for the heat transfer process during contact and offers some value in predicting the perceived temperature of any given material. Through continuous improvement and refinement, we can more accurately describe and predict various phenomena in this process.

Applicability and limitations

For analytical tractability, the model assumes temperature-independent thermophysical properties. Under the experimental conditions of this study, temperature excursions during contact are modest (typically < 10 K), over which variations in thermophysical properties are small compared to the orders-of-magnitude differences between material classes. Consequently, this approximation does not compromise the model's ability to support robust material discrimination.

The present model is applicable to short-duration thermo-tactile interactions at near-ambient temperatures, where heat transfer is predominantly one-dimensional. Under extreme conditions, such as large temperature excursions, strongly temperature-dependent material properties, prolonged contact times, or pronounced three-dimensional heat flow, more sophisticated numerical or temperature-dependent models would be required.

We emphasize that these limitations do not undermine the validity of the present results within the intended operating regime. Instead, the model provides a physically meaningful, experimentally validated framework for interpreting transient thermo-tactile signals and supporting high-accuracy material recognition.

Table S1. Thermal properties of nine common objects

Object	Thermal conductivity $\boldsymbol{k}$ (W/m·K)	Specific heat $\boldsymbol{c}$ (J/(kg·K))	Density $\boldsymbol{\rho}$ (kg/m^3)	Thermal contact coefficient $\sqrt{\boldsymbol{k\rho c}}$ (10^3J/(m^2·s$^{1/2}$·K))
Copper	398	384	8954	36992
Aluminum	237	900	2700	23998
Iron	60	460	7800	14672
Steel	13.5	460	8000	7048
Marble	2.79	775	2630	2384
Glass	1.38	652	2500	1500
PMMA	0.2	1386	1010	501
Wood	0.15	1670	500	354
Foam	0.029	1210	24	29

Note S3. Thermal conductivity measurement

As shown in fig. S9, the thermal conductivity of the i-TE skin was determined using a steady-state method. The measurement setup consists of two identical poly(methyl methacrylate) (PMMA) plates (thickness d = 5 mm, thermal conductivity (k_1, k_3) of 0.19 W m^{-1} K^{-1}) positioned in parallel on both sides of the i-TE skin, serving as heat transfer walls with a spacing of 5 mm. A Peltier heating unit was placed in contact with the left PMMA plate, while the i-TE skin with unknown thermal conductivity (k_2) and a cross-sectional area of 9 cm² (30 × 30 mm) was sandwiched between the PMMA plates.

To ensure equal input and output heat flux ($Q_{input} = Q_{ouput}$), the entire setup was embedded in thermal insulation foam to minimize heat loss. Under steady-state conditions, the heat flux across the PMMA plates is equal to that across the i-TE hydrogel, which can be expressed as:

$$\kappa_1 A \left(\frac{\partial T}{\partial d}\right)_1 = \kappa_2 A \left(\frac{\partial T}{\partial d}\right)_2 = \kappa_1 A \left(\frac{\partial T}{\partial d}\right)_3 \tag{13}$$

$$\kappa_2 = \kappa_1 \frac{\left(\frac{\partial T}{\partial d}\right)_1}{\left(\frac{\partial T}{\partial d}\right)_2} = \kappa_1 \frac{\left(\frac{\partial T}{\partial d}\right)_3}{\left(\frac{\partial T}{\partial d}\right)_2} \tag{14}$$

According to formula (13), if the steady-state temperature gradient ($\partial T/\partial d$) is achieved across the PMMA walls and hydrogel, then we can calculate the thermal conductivity of the hydrogel. The temperature changes for the hydrogel and PMMA walls were monitored using thermocouples (USB-TC-08, Pico Technology, St. Neots, UK). For each measurement, the device was incubated for a sufficient time (>1 h) to ensure that a steady temperature gradient had been built.

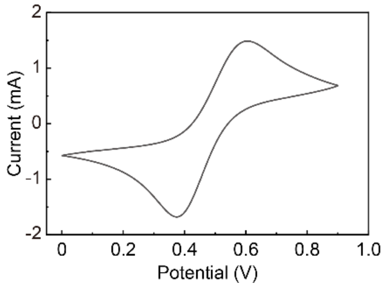

Figure S2. CV curve of the Fe^{2+}/Fe^{3+} redox couples.

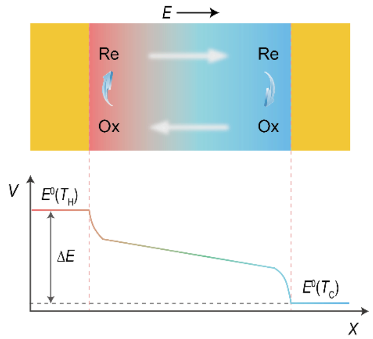

Figure S3. Schematic diagram of the thermogalvanic effect. It depicts the potential of charge carriers and the voltage distribution corresponding to reversible redox reactions, where *E* represents the built-in electric field.

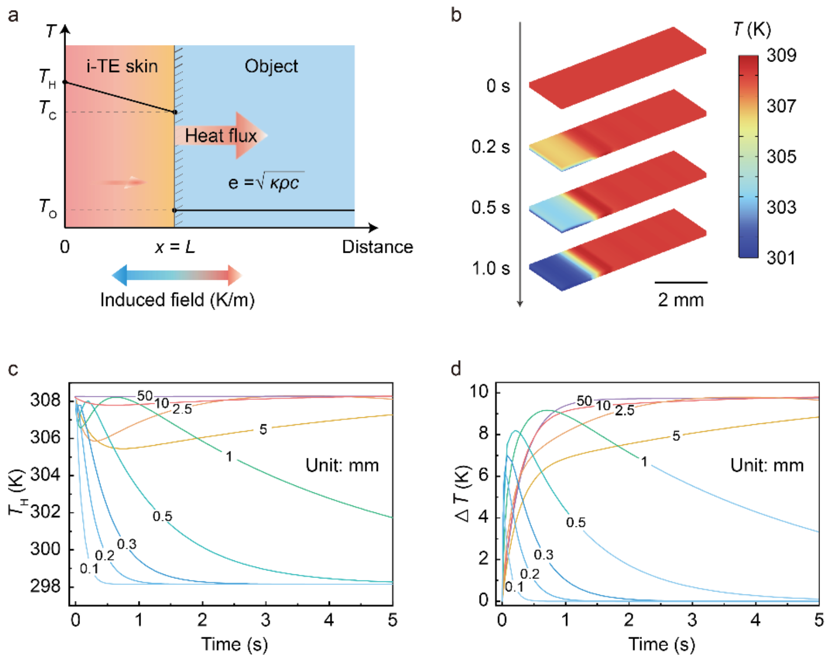

Figure S4. Temporal evolution of the temperature of the i-TE skin during thermal interaction with a steel cube. a) Schematic diagram of the thermal interaction between the i-TE skin and object. The shadow line represents the contact interface. The initial temperature in the single-layer model of i-TE skin with a thickness of L. At $x = 0$, the electrode end with a heat source maintains a fixed temperature of T_H. The electrode at the contact end T_C is located at $x = L$ with an initial temperature of T_{s0}. The external object is considered a semi-infinite body with an initial temperature of T_o. b) Temperature variations during the thermal interaction process between the i-TE skin and object. c) Temporal evolution of the temperature at the distal boundary (T_H) of the i-TE skin. d) The temperature difference ($T_H - T_C$) of the i-TE skin during the thermal contact process.

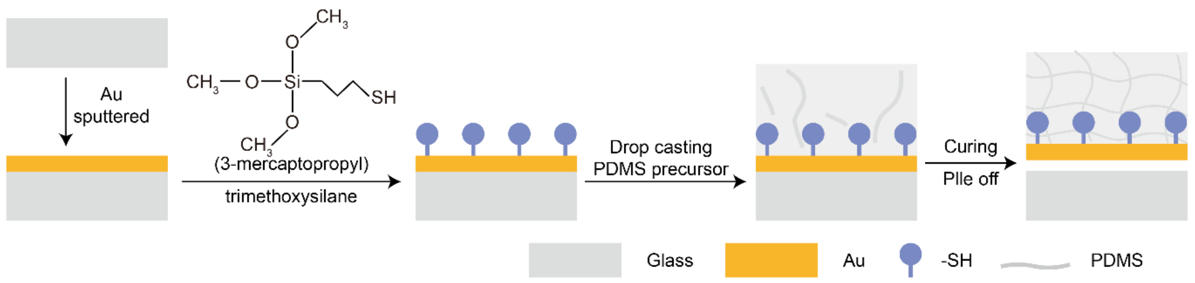

Figure S5. Preparation and electrical characterization of Au/PDMS electrodes. a) Schematic illustration of the preparation process for Au/PDMS flexible electrodes. A gold film is deposited onto a cleaned glass substrate using magnetron sputtering. The gold film surface is then modified with a self-assembled monolayer of (3-Mercaptopropyl)trimethoxysilane (MPTMS), forming strong gold-sulfur bonds. Covalent bonds are established when the modified gold film surface is brought into contact with the surface of the PDMS material. After curing the PDMS, the electrode is peeled off to obtain the Au/PDMS electrode.

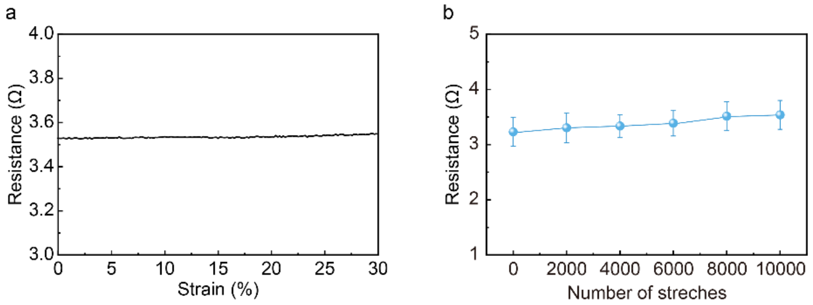

Figure S6. Mechanical stability of the Au/PDMS electrode. a) The resistance changes of the Au/PDMS electrode during a single instance of 30% tensile stretching. b) The resistance changes of the electrode over 10000 cycles of 30% tensile stretching and releasing.

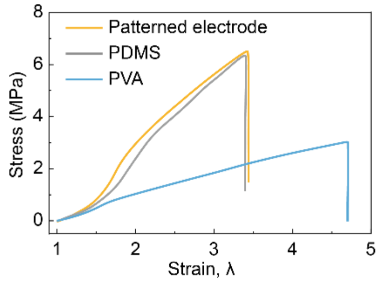

Figure S7. Stress-stretch curves of the patterned electrode, pure PDMS and $Fe^{2+/3+}$/PVA gel. The Young's modulus of the gel (0.78 MPa) is close to that of the patterned electrode (0.91 MPa) and PDMS (0.88 MPa).

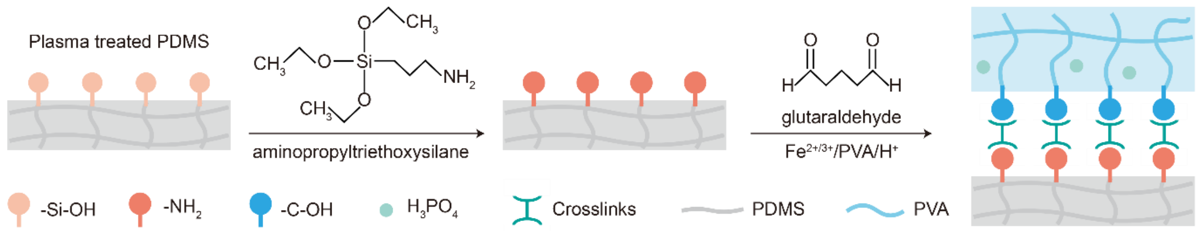

Figure S8. Schematic representation of the crosslinked interface between the $Fe^{2+/3+}$/PVA hydrogel and PDMS matrix.

Table S2. Comparison between temperature coefficient, chemical stability, reversibility, and hydrogel compatibility

Typical redox couples	Temperature coefficient (mV K^{-1})	Electrochemical reversibility	Stability in acidic condition	Compatible with PVA	Ref.
$Fe(CN)_6^{4-}/Fe(CN)_6^{3-}$	-1.4	Excellent	Unstable	×	58
$Co(bpy)_3^{2+}/Co(bpy)_3^{3+}$	1.6	Excellent	Stable	×	59
I^-/I_3^-	0.53	Excellent	Stable	√	60
Cu/Cu^{2+}	0.7	Excellent	Stable	√	61
Fe^{2+}/Fe^{3+}	1.24	Excellent	Stable	√	This work

Building on this material choice, we further clarified the mechanisms responsible for the excellent stability of the i-TE skin. Specifically, the robustness of the Fe^{2+}/Fe^{3+} system arises from (i) highly reversible and fast redox kinetics that suppress irreversible side reactions during repeated thermal cycling, (ii) thermodynamic stability of the redox equilibrium under acidic hydrogel fabrication and operating conditions, and (iii) confinement of redox ions within a cross-linked PVA hydrogel network combined with chemically bonded PDMS encapsulation, which together establish a stable electrochemical microenvironment and mitigate interfacial degradation under prolonged thermal and mechanical loading. Additionally, the strategic integration of additives, such as LiCl for moisture retention and glycerol for anti-freeze properties, further enhances the overall stability and reliability of the i-TE skin in the harsh environment. The physicochemical stability of the redox system, device architecture, and environmental adaptability of the hydrogel electrolyte collectively ensure mechanical integrity and electrochemical stability, supporting the suitability of the i-TE skin for long-term operation in practical thermo-tactile human–machine interfaces.

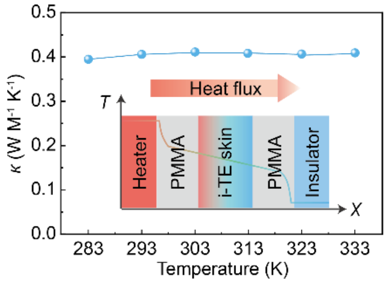

Figure S9. The fluctuation of the thermal conductivity at different temperatures.

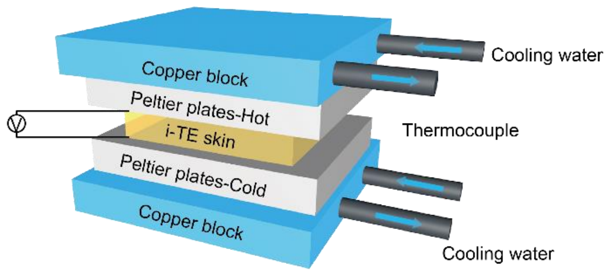

Figure S10. Thermoelectric measurement device for electric performance evaluation of the thermogalvanic hydrogels.

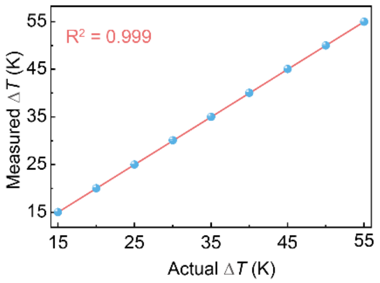

Figure S11. Performance of the i-TE skin for perceiving absolute temperature signals compared with commercial thermocouples.

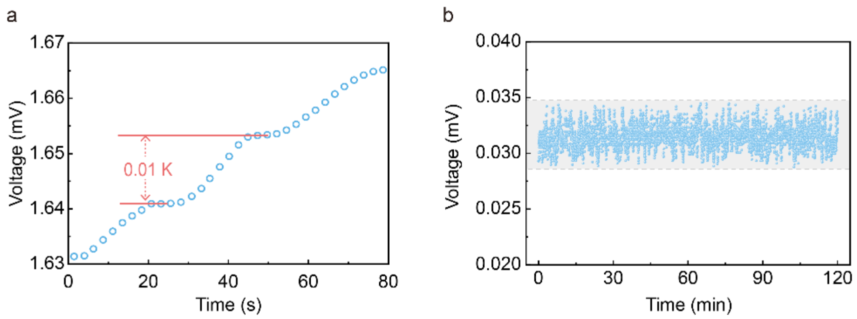

Figure S12. The thermal sensing performance of the i-TE skin. a) Voltage response time evolution curves of the i-TE skin. b) The temperature variation of the device measured in a constant temperature and humidity container.

The detection limit was calculated by determining the standard deviation of voltage fluctuations after placing the sensor in a constant temperature and humidity chamber for 2 hours. The standard deviations of the temperatures recorded by the i-TE skin were 0.81 mK.

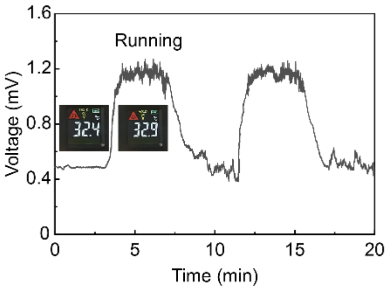

Figure S13. Continuous and minute temperature change monitoring by the i-TE skin during human motion.

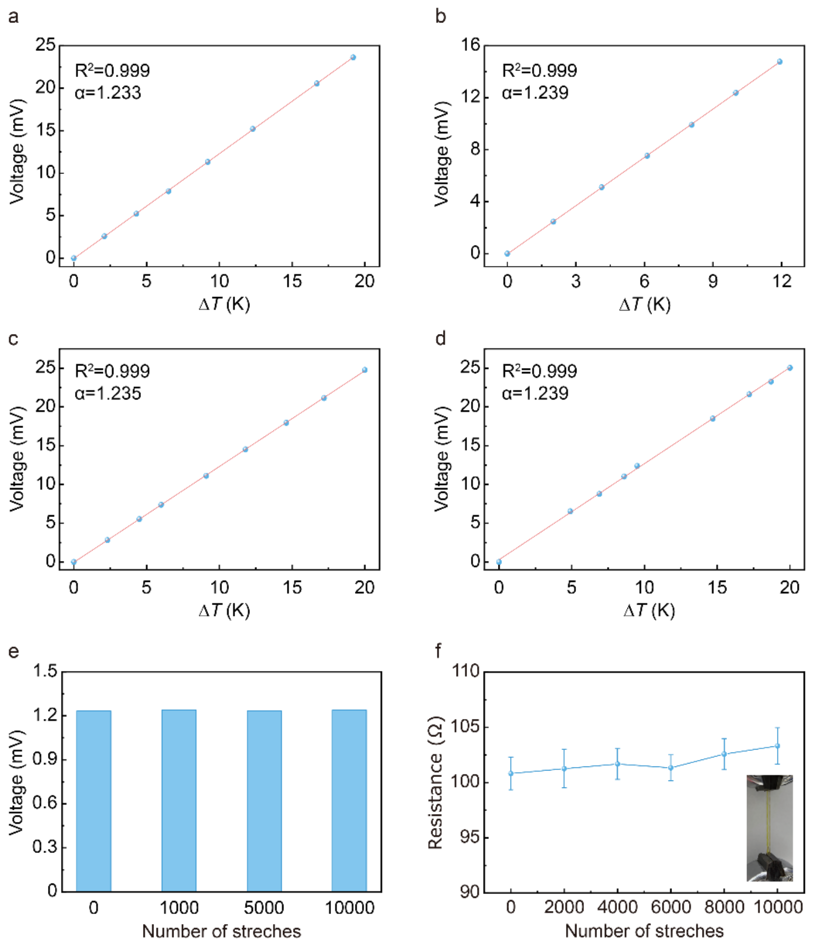

Figure S14. Electrical stability Characterization of the i-TE skin. The temperature coefficient of the i-TE skin before (a) and after 1000 (b), 5000 (c),10000 (d) operation cycles at different temperatures. (e) The temperature coefficient of the i-TE skin before and after 10000 operation cycles. f) The resistance changes of the sensor over 10000 cycles of 30% tensile stretching and releasing.

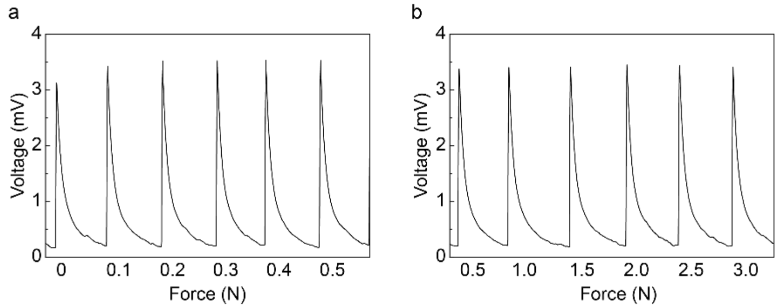

Figure S15. The output voltage of the i-TE skin under different pressure conditions. a) Voltage variation of the i-TE skin in the low-pressure range (0–0.5 N). b) Output stability in the practical pressure range (0.5–3.0 N).

The results indicate that within the pressure range of 0–0.5 N, as the pressure increases, the contact thermal resistance gradually decreases, thereby enhancing the thermal conduction efficiency and causing the output voltage to show an increasing trend. Considering that the common contact pressure in actual thermal interaction processes is usually between 1 and 3 N, the response characteristics of the i-TE skin within the pressure range of 0.5–3.0 N were further tested. The output voltage remains stable in this region, demonstrating that the i-TE skin maintains consistent thermal sensing performance and is largely insensitive to variations in applied pressure under realistic operational conditions.

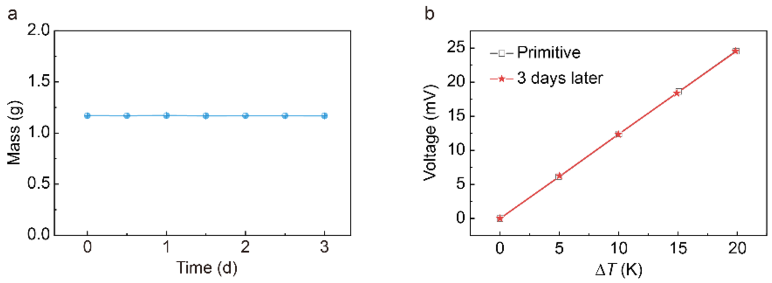

Figure S16. The long-term stability of the i-TE skin under elevated-temperature drying conditions. a) Mass evolution of the i-TE skin during accelerated aging at 60°C and 40% relative humidity for 72 hours. b) The temperature coefficient measured before and after the thermal aging test, showing negligible performance degradation.

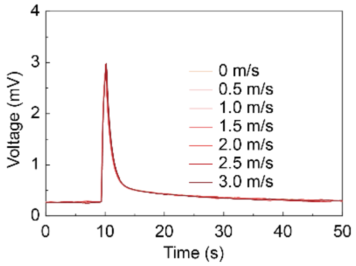

Figure S17. Voltage of the i-TE skin during contact with glass under different wind speeds.

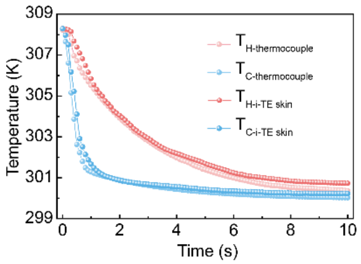

Figure S18. Temperature variation of the i-TE skin and the commercial thermocouples.

The thermocouples were attached directly to the surface of the i-TE skin. During material-contact experiments, the thermocouple's temperature readings and the i-TE skin's voltage output were recorded simultaneously. Using the experimentally established linear relationship between output voltage and temperature difference, the voltage data were converted into equivalent temperature values for direct comparison with the thermocouple readings.

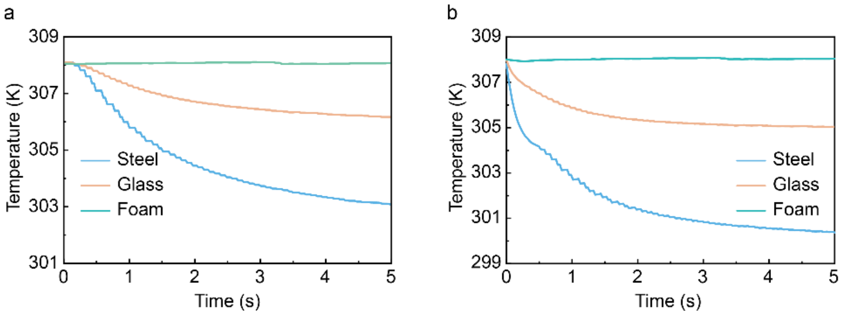

Figure S19. The thermal interaction between the i-TE skin and objects with different thermal properties. (a-b) Temperature variation of i-TE skin during the contact process. The temperature changes of the electrodes at the upper (a) and lower ends (b) when in contact with three kinds of objects with different thermal contact coefficients.

When in contact with low thermal contact coefficient objects, such as foam, the i-TE skin exhibited smaller temperature fluctuations, mainly due to the lower heat storage and transfer capabilities of these materials. Conversely, when in contact with high thermal contact coefficient objects, like metal, the temperature changes were more significant, as these high thermal contact coefficient materials can absorb or release heat more efficiently. This difference not only reveals the thermal properties of different materials but also further validates the sensitivity and response capability of the i-TE skin to the thermal attributes of various materials.

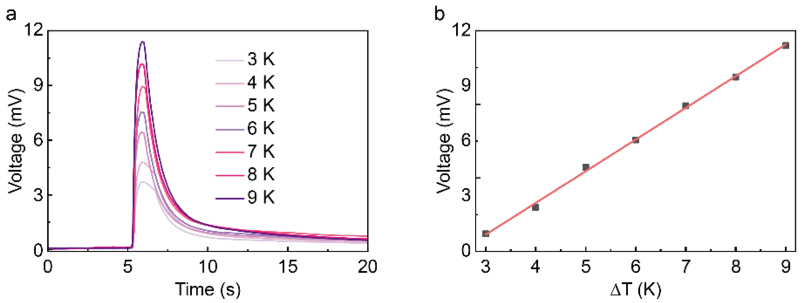

Figure S20. The relationship between the peak voltage of the i-TE skin and the temperature difference. a) The peak voltage of the sensor under different temperature differences. b) The linear relationship between temperature difference and peak voltage.

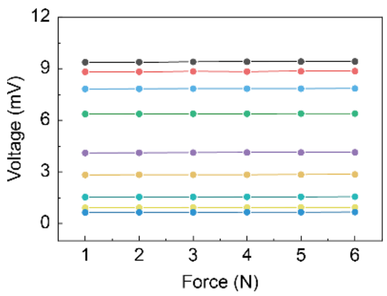

Figure S21. Voltage signals of i-TE skin under different pressures. Points of different colors represent data sets of different materials. The color sequence is consistent with that in Fig. 4A.

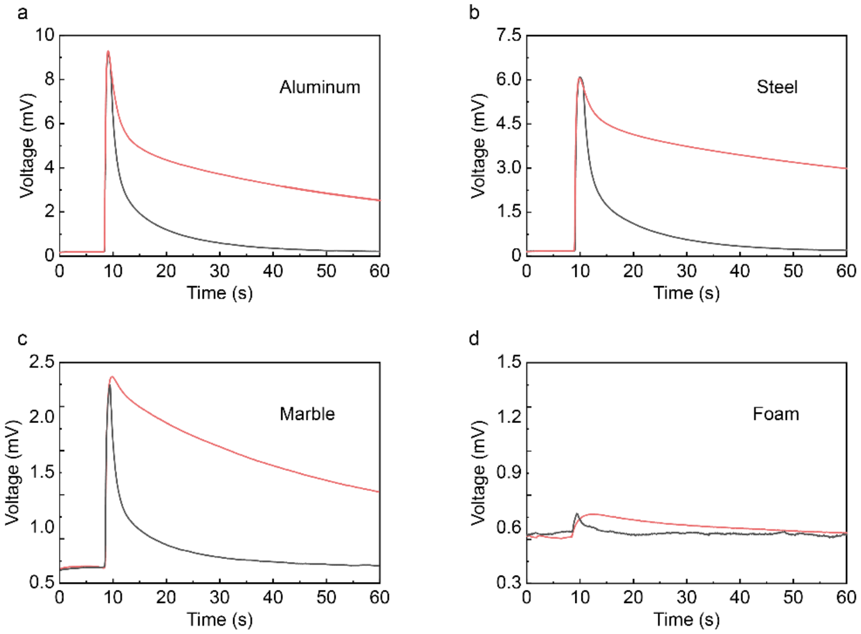

Figure S22. Voltage output of the i-TE skin for four objects at different contact frequencies.

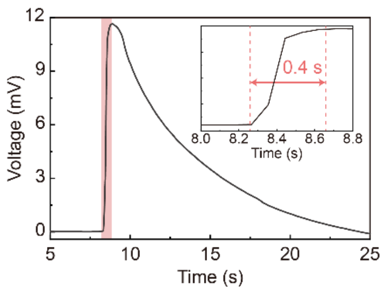

Figure S23. Response speed of the sensor when in contact with copper at room temperature. The response time is defined as the time required to reach 90% of the maximum voltage change.

Table S3. Comparison between a conventional heat-flux sensor and the i-TE skin

Aspect	i-TE skin (this work)	Conventional heat-flux sensors
**Operating mechanism**	Thermogalvanic effect based on reversible redox reactions	Thermopile-based or Seebeck-effect-based electronic thermoelectrics
**Primary sensing quantity**	Time-resolved voltage encoding transient heat flux	Steady-state or averaged heat flux
**Temporal response**	Intrinsically sensitive to transient thermal events upon contact	Typically optimized for steady or slowly varying heat flux
**Mechanical properties**	Soft, flexible, skin-conformable hydrogel-based device	Generally rigid, bulky substrates

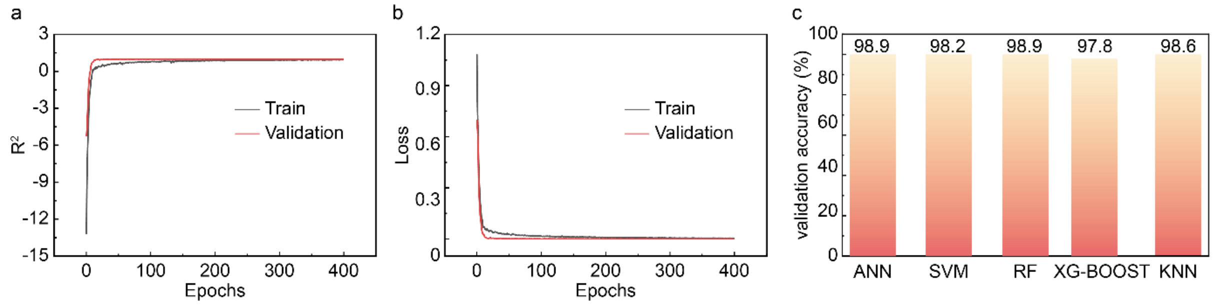

Figure S24. Machine learning for material recognition. (a-b) The optimization of the validation set. c) Recognition accuracy statistics of different algorithms.

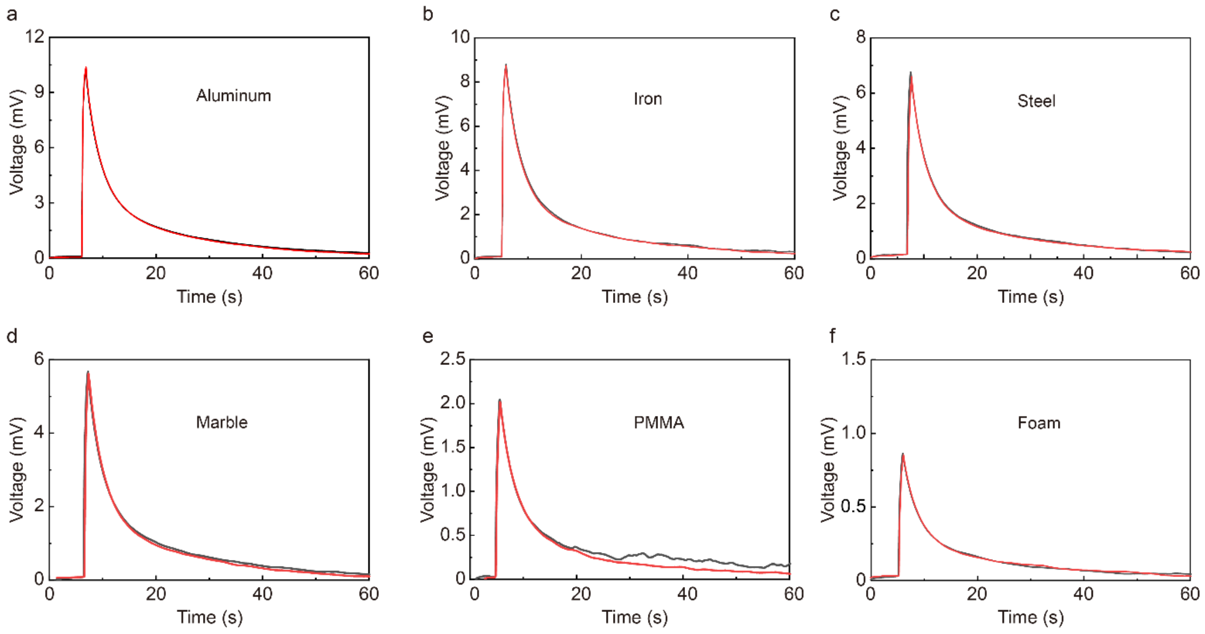

Figure S25. The voltage response of the i-TE skin contact with the surface of an object before (black curve) and after (red curve) being blackened. Figures (a-f) are aluminum (a), iron (b), steel (c), marble (d), PMMA (e), and foam (f).

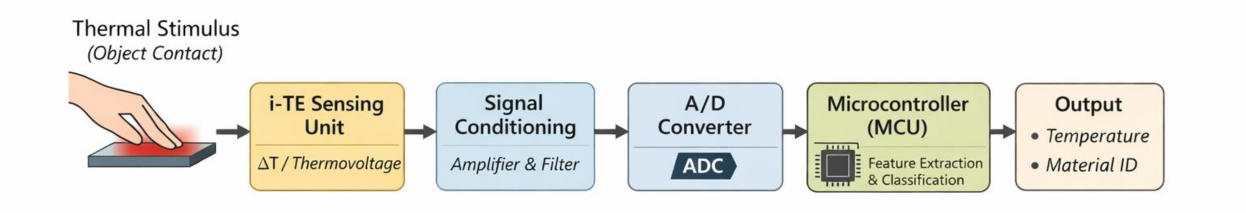

Figure S26. Signal processing circuit of the system.

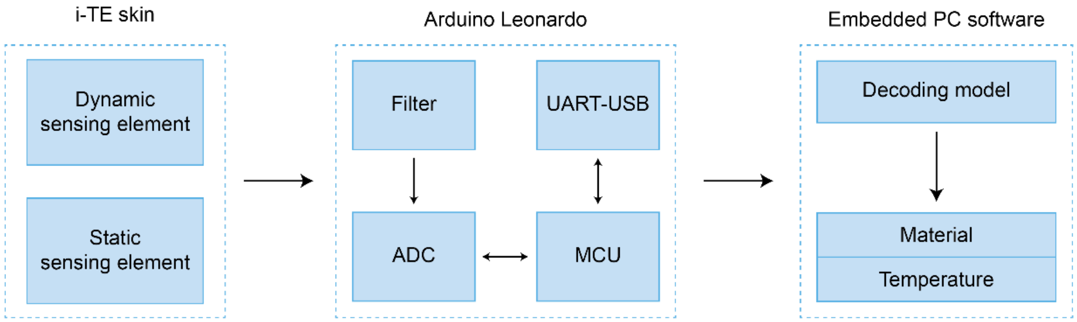

Figure S27. A schematic diagram of the system illustrating the workflow of signal generation, processing, and transmission. The i-TE skin detects both transient heat flux (dynamic sensing element) and quasi-steady temperature (static sensing element) information and converts them into electrical signals. The raw signals are filtered to suppress noise and improve signal stability, then digitized and processed by a microcontroller unit (MCU). In real-time operation, characteristic features are extracted from each single contact event and transmitted via USB to a mobile device, where a pre-trained ANN performs event-driven material classification. This pipeline enables real-time material recognition while preserving independent access to dynamic material-specific signatures and static temperature information.

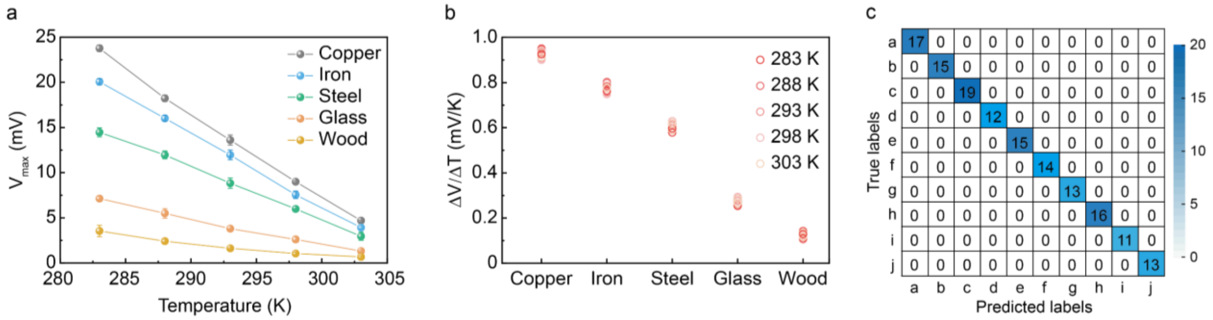

Figure S28. The i-TE skin contacts different objects at different temperatures. a) The temperature and voltage characteristics of different objects under varied initial conditions. b) Normalized initial slope illustrating material-specific clustering independent of ΔT. c) Confusion matrix of the ANN model validation set. Each material was tested 100 times.

To experimentally validate this assumption, we conducted additional measurements in which material samples (copper, stainless steel, glass, and wood) of identical size were pre-conditioned to different initial temperatures (25°C and 15°C) before contact with the i-TE skin. Each material was tested 100 times. The numbers a–j in fig. S28 corresponds respectively to the test samples of the five materials under the above conditions at two temperatures. The resulting transient voltage responses were used to train and validate an artificial neural network.

Note S4. Comparison with reported electronic skin systems

Over the past decade, electronic skin (e-skin) has advanced rapidly, integrating novel materials, device engineering, and sensing modalities to achieve various haptic perceptions. To provide realistic tactile feedback, it is essential to reproduce diverse tactile sensations, mirroring how our brain processes a wealth of sensory information to experience the world. Specifically, humans can identify materials like metal or wood not just by their surface characteristics but also by their perceived coolness or warmth upon contact. However, thermo-tactile sensing, a critical aspect of haptic feedback, remains largely underexplored. Most conventional robotic systems and e-skin primarily focus on pressure or strain detection and rely heavily on mechanical haptic interfaces or machine vision for material identification. Moreover, most existing thermal devices are limited to static temperature measurements, unable to achieve material recognition through thermal perception.

To address these limitations, our i-TE skin provides modality-specific thermal feedback while operating autonomously under small temperature gradients. This approach avoids the high power consumption, risk of mechanical failure, and complex actuation requirements associated with conventional electroactive systems. By integrating thermogalvanic sensing elements with anisotropic thermal response characteristics, the device can simultaneously detect temperature, identify material, and perceive thermal hazards. Its design mimics the diverse activation patterns of human thermoreceptors, providing enhanced perception of surface properties and rich thermal feedback. Our study aims to gain a comprehensive understanding of the relationship between stimulation patterns and thermal perception, thereby enabling richer virtual haptic worlds with integrated functions. Representative works that have combined different types of stimulation to provide multimodal haptic feedback are summarized in table S4.

Table S4. Comparison of our work with recent published sensors

Types	Demonstration	Sensing mechanisms	Perception	Configuration engineering	Ref.
Thermal	Wearable device	Thermogalvanic effect	Temperature and material identification	Seamless integration strategy	This work
	Thermal sensor arrays	Ion migration influenced by temperature	Temperature	Encapsulated by UV-cured resin	21
	Wearable glove	Heating wires	Temperature, vibration	Encapsulated by soft elastomers	62
	Stretchable patch	Thermoelectric pellets	Temperature, cold and warm sensations	Selective thermal engineering	36
	Distributed mini-region sensing	Thermoelectric generators	Temperature	Island-bridge structure	63
	Thermoelectric paster	REDOX effect and Soret effect	Temperature, material identification	Sandwiched structure	64
	Wearable device	Asymmetric ion distribution	Temperature, material identification	Stacked configuration	65
	Wearable device	Relaxation behavior of ionic gels	Temperature, pressure, and proximity	Sandwiched structure	14
	Thermal Sensor arrays	Soret effect	Temperature	Stacked configuration	13
Integration of two kinds	Wearable device	Thermal resistance effect, tribo-electrode effect	Temperature, material identification	Stacked configuration	66
	Wearable Device	Thermal resistance effect and logical reasoning	Temperature, material identification and pressure	Stacked configuration	67
Mechanical	Wearable Device	Capacitance effect	Pressure and material identification	Embedded configuration	68
	Wearable Device	Piezoionic effect	Material identification	Stacked configuration	69
	Wearable Device	Triboelectric effect	Material identification	Stacked configuration	34

Movie S1. Real-time thermo-tactile material recognition using the i-TE skin integrated on a robotic fingertip

REFERENCES

1. F. Iberite, J. Muheim, O. Akouissi, S. Gallo, G. Rognini, F. Morosato, A. Clerc, M. Kalff, E. Gruppioni, S. Micera, S. Shokur, Restoration of natural thermal sensation in upper-limb amputees. *Science* **380**, 731–735 (2023).

2. X. Fu, W. Cheng, G. Wan, Z. Yang, B. C. K. Tee, Toward an AI era: Advances in electronic skins. *Chem. Rev.* **124**, 9899–9948 (2024).

3. A. Dhaka, V. Viswanath, A. Patapoutian, Trp ion channels and temperature sensation. *Annu. Rev. Neurosci.* **29**, 135–161 (2006).

4. D. Julius, TRP channels and pain. *Annu. Rev. Cell Dev. Biol.* **29**, 355–384 (2013).

5. M. Paré, C. Dugas, Developmental changes in prehension during childhood. *Exp. Brain Res.* **125**, 239–247 (1999).

6. M. Vestergaard, M. Carta, G. Güney, J. F. A. Poulet, The cellular coding of temperature in the mammalian cortex. *Nature* **614**, 725–731 (2023).

7. M. Carta, M. Vestergaard, J. F. A. Poulet, The neuronal circuits and cellular encoding of thermosensation. *Nat. Rev. Neurosci.* **27**, 219–235 (2026).

8. L. Qi, M. Iskols, D. Shi, P. Reddy, C. Walker, K. Lezgiyeva, T. Voisin, M. Pawlak, V. K. Kuchroo, I. M. Chiu, D. D. Ginty, N. Sharma, A mouse DRG genetic toolkit reveals morphological and physiological diversity of somatosensory neuron subtypes. *Cell* **187**, 1508–1526.e16 (2024).

9. R. Xiao, X. Z. S. Xu, Temperature sensation: From molecular thermosensors to neural circuits and coding principles. *Annu. Rev. Physiol.* **83**, 205–230 (2021).

10. D. Filingeri, Neurophysiology of skin thermal sensations. *Compr. Physiol.* **6**, 1429–1491 (2016).

11. J. Vriens, B. Nilius, T. Voets, Peripheral thermosensation in mammals. *Nat. Rev. Neurosci.* **15**, 573–589 (2014).

12. R. J. Schepers, M. Ringkamp, Thermoreceptors and thermosensitive afferents. *Neurosci. Biobehav. Rev.* **34**, 177–184 (2010).

13. Y. Han, H. Wei, Y. Du, Z. Li, S.-P. Feng, B. Huang, D. Xu, Ultrasensitive flexible thermal sensor arrays based on high-thermopower ionic thermoelectric hydrogel. *Adv. Sci.* **10**, e2302685 (2023).

14. W. Sun, P. Zhang, X. Lin, Y. Wang, S. Wang, B. Yang, Z. Zheng, W. Liu, Heat source recognition sensor mimicking the thermosensation function of human skin. *Int. J. Hydrogen Energ.* **5**, 100673 (2024).

15. K. K. Kim, M. Kim, K. Pyun, J. Kim, J. Min, S. Koh, S. E. Root, J. Kim, B.-N. T. Nguyen, Y. Nishio, S. Han, J. Choi, C.-Y. Kim, J. B.-H. Tok, S. Jo, S. H. Ko, Z. Bao, A substrate-less nanomesh receptor with meta-learning for rapid hand task recognition. *Nat. Electron.* **6**, 64–75 (2023).

16. K. K. Kim, I. Ha, M. Kim, J. Choi, P. Won, S. Jo, S. H. Ko, A deep-learned skin sensor decoding the epicentral human motions. *Nat. Commun.* **11**, 2149 (2020).

17. Y. Dobashi, D. Yao, Y. Petel, T. N. Nguyen, M. S. Sarwar, Y. Thabet, C. L. W. Ng, E. Scabeni Glitz, G. T. M. Nguyen, C. Plesse, F. Vidal, C. A. Michal, J. D. W. Wadden, Piezoionic mechanoreceptors: Force-induced current generation in hydrogels. *Science* **376**, 502–507 (2022).

18. I. You, D. G. Mackanic, N. Matsuhisa, J. Kang, J. Kwo, L. Beker, J. Mun, W. Suh, T. Y. Kim, J. B.-H. Tok, Z. Bao, Artificial multimodal receptors based on ion relaxation dynamics. *Science* **370**, 961–965 (2020).

19. R. Di Giacomo, L. Bonanomi, V. Costanza, B. Maresca, C. Daraio, Biomimetic temperature-sensing layer for artificial skins. *Sci. Robot.* **2**, eaai9251 (2017).

20. X. Liu, X. Ji, R. Zhu, J. Gu, J. Liang, A microphase-separated design toward an all-round ionic hydrogel with discriminable and anti-disturbance multisensory functions. *Adv. Mater.* **36**, e2309508 (2024).

21. J. Zhang, K. Yan, J. Huang, X. Sun, J. Li, Y. Cheng, Y. Sun, Y. Shi, L. Pan, Mechanically robust, flexible, fast responding temperature sensor and high-resolution array with ionically conductive double cross-linked hydrogel. *Adv. Funct. Mater.* **34**, 2314433 (2024).

22. Y.-E. Shin, Y.-J. Park, S. K. Ghosh, Y. Lee, J. Park, H. Ko, Ultrasensitive multimodal tactile sensors with skin-inspired microstructures through localized ferroelectric polarization. *Adv. Sci.* **9**, e2105423 (2022).

23. Y. Chen, H. Lei, Z. Gao, J. Liu, F. Zhang, Z. Wen, X. Sun, Energy autonomous electronic skin with direct temperature-pressure perception. *Nano Energy* **98**, 107273 (2022).

24. Y. Fu, S. Kang, G. Xiang, C. Su, C. Gao, L. Tan, H. Gu, S. Wang, Z. Zheng, S. Dai, C. Lin, Ultraflexible temperature-strain dual-sensor based on chalcogenide glass-polymer film for human-machine interaction. *Adv. Mater.* **36**, e2313101 (2024).

25. K. K. Kim, J. Bang, M. Kim, J. Jeong, I. Ha, S. H. Ko, Unisensory processing of interleaving memristive nanowires enabling multimodal sensing at human-scale resolution. *Nat. Mater.* **25**, 463–471 (2026).

26. X. Zhang, J. Chen, Z. Zheng, S. Tang, B. Cheng, Z. Zhang, R. Ma, Z. Chen, J. Zhuo, L. Cao, Z. Chen, J. He, X. Wang, G. Yang, F. Yi, Flexible temperature sensor with high reproducibility and wireless closed-loop system for decoupled multimodal health monitoring and personalized thermoregulation. *Adv. Mater.* **36**, e2407859 (2024).

27. J. Wu, Z. Wu, Y. Wei, H. Ding, W. Huang, X. Gui, W. Shi, Y. Shen, K. Tao, X. Xie, Ultrasensitive and stretchable temperature sensors based on thermally stable and self-healing organohydrogels. *ACS Appl. Mater. Interfaces* **12**, 19069–19079 (2020).

28. J. Shin, B. Jeong, J. Kim, V. B. Nam, Y. Yoon, J. Jung, S. Hong, H. Lee, H. Eom, J. Yeo, J. Choi, D. Lee, S. H. Ko, Sensitive wearable temperature sensor with seamless monolithic integration. *Adv. Mater.* **32**, e1905527 (2020).

29. J. Neto, R. Chirila, A. S. Dahiya, A. Christou, D. Shakthivel, R. Dahiya, Skin-inspired thermoreceptors-based electronic skin for biomimicking thermal pain reflexes. *Adv. Sci.* **9**, e2201525 (2022).

30. G. Li, S. Liu, L. Wang, R. Zhu, Skin-inspired quadruple tactile sensors integrated on a robot hand enable object recognition. *Sci. Robot.* **5**, eabc8134 (2020).

31. J. Wade, T. Bhattacharjee, R. D. Williams, C. C. Kemp, A force and thermal sensing skin for robots in human environments. *Rob. Auton. Syst.* **96**, 1–14 (2017).

32. X. Wei, B. Wang, Z. Wu, Z. L. Wang, An open-environment tactile sensing system: Toward simple and efficient material identification. *Adv. Mater.* **34**, e2203073 (2022).

33. W. Xiong, F. Zhang, S. Qu, L. Yin, K. Li, Y. Huang, Marangoni-driven deterministic formation of softer, hollow microstructures for sensitivity-enhanced tactile system. *Nat. Commun.* **15**, 5596 (2024).

34. S. He, J. Dai, D. Wan, S. Sun, X. Yang, X. Xia, Y. Zi, Biomimetic bimodal haptic perception using triboelectric effect. *Sci. Adv.* **10**, eado6793 (2024).

35. F. Li, H. Xue, X. Lin, J. Wang, J. Li, H. Zhao, T. Zhang, Energy landscape–engineered iontronics enable artificial thermoreceptors for augmented bioinspired thermosensation. *Sci. Adv.* **11**, eady2547 (2025).

36. J. Lee, H. Sul, W. Lee, K. R. Pyun, I. Ha, D. Kim, H. Park, H. Eom, Y. Yoon, J. Jung, D. Lee, S. H. Ko, Stretchable skin-like cooling/heating device for reconstruction of artificial thermal sensation in virtual reality. *Adv. Funct. Mater.* **30**, 1909171 (2020).

37. B. Yu, J. Duan, H. Cong, W. Xie, R. Liu, X. Zhuang, H. Wang, B. Qi, M. Xu, Z. L. Wang, J. Zhou, Thermosensitive crystallization–boosted liquid thermocells for low-grade heat harvesting. *Science* **370**, 342–346 (2020).

38. C.-G. Han, X. Qian, Q. Li, B. Deng, Y. Zhu, Z. Han, W. Zhang, W. Wang, S.-P. Feng, G. Chen, W. Liu, Giant thermopower of ionic gelatin near room temperature. *Science* **368**, 1091–1098 (2020).

39. L. Liu, X. Guo, D. Zhang, R. Ma, Thermogalvanic hydrogels for low-grade heat harvesting and health monitoring. *Mater. Horiz.* **12**, 5473–5491 (2025).

40. Y. Zeng, B. Yu, M. Chen, J. Zhang, P. Liu, J. Guo, J. Wang, G. Feng, J. Zhou, J. Duan, Solvation entropy engineering of thermogalvanic electrolytes for efficient electrochemical refrigeration. *Joule* **9**, 101822 (2025).

41. A. Rajan, I. S. McKay, S. K. Yee, Continuous electrochemical refrigeration based on the Brayton cycle. *Nat. Energy* **7**, 320–328 (2022).

42. B. Yu, Z. L. Wang, J. Duan, Entropy engineering for efficient ionic thermoelectric conversion. *Natl. Sci. Rev.*, https://doi.org/10.1093/nsr/nwag031 (2026).

43. M. F. Dupont, D. R. MacFarlane, J. M. Pringle, Thermo-electrochemical cells for waste heat harvesting—Progress and perspectives. *Chem. Commun.* **53**, 6288–6302 (2017).

44. W. Gao, Z. Lei, C. Zhang, X. Liu, Y. Chen, Stretchable and freeze-tolerant organohydrogel thermocells with enhanced thermoelectric performance continually working at subzero temperatures. *Adv. Funct. Mater.* **31**, 2104071 (2021).

45. A. Dufour, O. Després, T. Pebayle, S. Lithfous, Thermal sensitivity in humans at the depth of thermal receptor endings beneath the skin: Validation of a heat transfer model of the skin using high-temporal resolution stimuli. *Eur. J. Appl. Physiol.* **120**, 1509–1518 (2020).

46. A. F. Mills, *Heat Transfer* (Irwin, 1992).

47. Y. Liu, G. Guan, Y. Li, J. Tan, P. Cheng, M. Yang, B. Li, Q. Wang, W. Zhong, K. Mequanint, Z. Chuhong, X. Malcolm, Gelation of highly entangled hydrophobic macromolecular fluid for ultrastrong underwater in situ fast tissue adhesion. *Sci. Adv.* **8**, eabm9744 (2022).

48. A. Inoue, H. Yuk, B. Lu, X. Zhao, Strong adhesion of wet conducting polymers on diverse substrates. *Sci. Adv.* **6**, eaay5394 (2020).

49. C. Lim, Y. J. Hong, J. Jung, Y. Shin, S.-H. Sunwoo, S. Baik, O. K. Park, S. H. Choi, T. Hyeon, J. H. Kim, S. Lee, D.-H. Kim, Tissue-like skin-device interface for wearable bioelectronics by using ultrasoft, mass-permeable, and low-impedance hydrogels. *Sci. Adv.* **7**, eabd3716 (2021).

50. H. Yuk, T. Zhang, S. Lin, G. A. Parada, X. Zhao, Tough bonding of hydrogels to diverse non-porous surfaces. *Nat. Mater.* **15**, 190–196 (2016).

51. Q. Liu, G. Nian, C. Yang, S. Qu, Z. Suo, Bonding dissimilar polymer networks in various manufacturing processes. *Nat. Commun.* **9**, 846 (2018).

52. Y. Shin, H. S. Lee, Y. J. Hong, S.-H. Sunwoo, O. K. Park, S. H. Choi, D.-H. Kim, S. Lee, Low-impedance tissue-device interface using homogeneously conductive hydrogels chemically bonded to stretchable bioelectronics. *Sci. Adv.* **10**, eadi7724 (2024).

53. P. Yang, K. Liu, Q. Chen, X. Mo, Y. Zhou, S. Li, G. Feng, J. Zhou, Wearable thermocells based on gel electrolytes for the utilization of body heat. *Angew. Chem. Int. Ed. Engl.* **55**, 12050–12053 (2016).

54. M. J. Caterina, T. A. Rosen, M. Tominaga, A. J. Brake, D. Julius, A capsaicin-receptor homologue with a high threshold for noxious heat. *Nature* **398**, 436–441 (1999).

55. D. M. Bautista, J. Siemens, J. M. Glazer, P. R. Tsuruda, A. I. Basbaum, C. L. Stucky, S.-E. Jordt, D. J. N. Julius, The menthol receptor TRPM8 is the principal detector of environmental cold. *Nature* **448**, 204–208 (2007).

56. S. Oss, A simple model of thermal conduction in human skin: Temperature perception and thermal effusivity. *Eur. J. Phys.* **43**, 035101 (2022).

57. S. Agustín, On thermal diffusivity. *Eur. J. Phys.* **24**, 351 (2003).

58. H. Im, T. Kim, H. Song, J. Choi, J. S. Park, R. Ovalle-Robles, H. D. Yang, K. D. Kihm, R. H. Baughman, H. H. Lee, T. J. Kang, Y. H. Kim, High-efficiency electrochemical thermal energy harvester using carbon nanotube aerogel sheet electrodes. *Nat. Commun.* **7**, 10600 (2016).

59. P. F. Salazar, S. T. Stephens, A. H. Kazim, J. M. Pringle, B. A. Cola, Enhanced thermo-electrochemical power using carbon nanotube additives in ionic liquid redox electrolytes. *J. Mater. Chem. A* **2**, 20676–20682 (2014).

60. A. Sosnowska, E. Laux, H. Keppner, T. Puzyn, M. Bobrowski, Relatively high-Seebeck thermoelectric cells containing ionic liquids supplemented by cobalt redox couple. *J. Mol. Liq.* **316**, 113871 (2020).

61. M. Owusu, K. Mensah-Darkwa, A. Andrews, F. Davis, P. Phelan, Effect of transient low-grade solar heat on liquid thermogalvanic cells. *Mater. Today Proc.* **38**, 767–772 (2021).

62. J. Oh, S. Kim, S. Lee, S. Jeong, S. H. Ko, J. Bae, A liquid metal based multimodal sensor and haptic feedback device for thermal and tactile sensation generation in virtual reality. *Adv. Funct. Mater.* **31**, 2007772 (2021).

63. M. Kang, R. Qu, X. Sun, Y. Yan, Z. Ma, H. Wang, K. Yan, W. Zhang, Y. Deng, Self-powered temperature electronic skin based on island-bridge structure and Bi-Te micro-thermoelectric generator for distributed mini-region sensing. *Adv. Mater.* **35**, e2309629 (2023).

64. Z. Du, L. Li, G. Shen, An ultra-thin wearable thermoelectric paster based on structured organic ion gel electrolyte. *Nano Micro Lett.* **17**, 204 (2025).

65. F. Li, X. Lin, H. Xue, J. Wang, J. Li, T. Fei, S. Liu, T. Zhou, H. Zhao, T. Zhang, Ultrasensitive flexible temperature sensors based on thermal-mediated ions migration dynamics in asymmetrical polymer bilayers. *ACS Nano* **18**, 7521–7531 (2024).

66. J. Chen, A. Liu, Y. Shi, Y. Luo, J. Li, M. Ye, W. Guo, Skin-inspired bimodal receptors for object recognition and temperature sensing simulation. *Adv. Funct. Mater.* **34**, 2403528 (2024).

67. Q. Mao, R. Zhu, Enhanced robotic tactile perception with spatiotemporal sensing and logical reasoning for robust object recognition. *Appl. Phys. Rev.* **11**, 021424 (2024).

68. J. Shi, Y. Dai, Y. Cheng, S. Xie, G. Li, Y. Liu, J. Wang, R. Zhang, N. Bai, M. Cai, Y. Zhang, Y. Zhan, Z. Zhang, C. Yu, C. F. Guo, Embedment of sensing elements for robust, highly sensitive, and cross-talk–free iontronic skins for robotics applications. *Sci. Adv.* **9**, eadf8831 (2023).

69. M. Ding, P. Xie, J. Wang, W. Guo, H. Li, S. Hu, D. Li, B. Li, N. Wang, C.-Y. Wong, J. Sun, J. C. Ho, Biomimetic microstructure design for ultrasensitive piezoionic mechanoreceptors in multimodal object recognition. *Nat. Commun.* **16**, 8129 (2025).
